# Systemic Metabolomic Profiling of Acute Myeloid Leukemia Patients before and During Disease-Stabilizing Treatment Based on All-Trans Retinoic Acid, Valproic Acid, and Low-Dose Chemotherapy

**DOI:** 10.3390/cells8101229

**Published:** 2019-10-10

**Authors:** Ida Sofie Grønningsæter, Hanne Kristin Fredly, Bjørn Tore Gjertsen, Kimberley Joanne Hatfield, Øystein Bruserud

**Affiliations:** 1 Department of Medicine, Haukeland University Hospital, 5021 Bergen, Norway; Ida.Gronningseter@uib.no (I.S.G.); Bjorn.Gjertsen@uib.no (B.T.G.); 2 Section for Hematology, Institute of Clinical Science, University of Bergen, 5021 Bergen, Norway; Kimberley.Hatfield@uib.no; 3 Department of Medicine, Bærum Hospital, 1346 Bærum, Norway; Harfre@vestreviken.no; 4 Department of Immunology and Transfusion Medicine, Haukeland University Hospital, 5021 Bergen, Norway

**Keywords:** acute myeloid leukemia, all-trans retinoic acid, lipids, metabolomics, valproic acid

## Abstract

Acute myeloid leukemia (AML) is an aggressive malignancy, and many elderly/unfit patients cannot receive intensive and potentially curative therapy. These patients receive low-toxicity disease-stabilizing treatment. The combination of all-trans retinoic acid (ATRA) and the histone deacetylase inhibitor valproic acid can stabilize the disease for a subset of such patients. We performed untargeted serum metabolomic profiling for 44 AML patients receiving treatment based on ATRA and valproic acid combined with low-dose cytotoxic drugs (cytarabine, hydroxyurea, 6-mercaptopurin) which identified 886 metabolites. When comparing pretreatment samples from responders and non-responders, metabolites mainly belonging to amino acid and lipid (i.e., fatty acid) pathways were altered. Furthermore, patients with rapidly progressive disease showed an extensively altered lipid metabolism. Both ATRA and valproic acid monotherapy also altered the amino acid and lipid metabolite profiles; however, these changes were only highly significant for valproic acid treatment. Twenty-three metabolites were significantly altered by seven-day valproic acid treatment (*p* < 0.05, *q* < 0.05), where the majority of altered metabolites belonged to lipid (especially fatty acid metabolism) and amino acid pathways, including several carnitines. These metabolomic effects, and especially the effects on lipid metabolism, may be important for the antileukemic and epigenetic effects of this treatment.

## 1. Introduction

Acute myeloid leukemia (AML) is an aggressive malignancy [1], and the only possible cure is intensive therapy possibly including stem cell transplantation [2]. The median age at first diagnosis is 65–70 years [3,4], and the elderly patients above 70–75 years of age have an increased frequency of high-risk disease [4] where remission is unlikely [5,6]. In addition, these patients are often not eligible to receive intensive treatment [7]. Furthermore, intensive chemotherapy is not always recommended for patients with refractory or relapsed disease either [8]. Such patients may instead receive low-toxicity AML-stabilizing treatment, e.g., a histone deacetylase (HDAC) inhibitor combined with all-trans retinoic acid (ATRA) and low-dose cytotoxic drugs [9].

HDAC inhibitors increase acetylation of various proteins, including histones [10], and this last effect increases gene transcription [11], especially in AML cells where HDACs often are overexpressed [12]. Valproic acid is a short-chain fatty acid that functions as an HDAC inhibitor [13] and has antiproliferative and proapoptotic effects in AML cells [14,15,16,17,18]. The toxicity of this drug is low, and its antileukemic activity alone or in combination with ATRA has been documented in several clinical studies [19,20]. ATRA is a derivative of vitamin A that binds to retinoid-responsive nuclear receptors [12,21]; it has antileukemic activity [22,23,24] and seems to increase the antileukemic effects of HDAC inhibitors [25,26]. Thus, the combination of ATRA and valproic acid seems to be a low-toxicity AML-stabilizing treatment; 30–40% of the patients respond to this therapy and responses may last up to one to two years [19,20]. This treatment can also be used in elderly and unfit patients [19,27]. Finally, AML cell metabolism is important for chemosensitivity in AML [28,29], and both ATRA and valproic acid can modulate cellular and/or systemic metabolism [30,31,32,33,34,35,36,37].

Even though the antileukemic in vivo effects of both ATRA and valproic acid are limited, both drugs should still be regarded as candidates for the treatment of non-acute promyelocytic leukemia (non-APL) variants of AML, especially in combination with other antileukemic agents in selected subsets of patients. First, although a large clinical study could not find a general improvement of overall survival when ATRA was added to intensive chemotherapy, increased survival was observed for subsets of patients, especially patients with a favorable prognosis (i.e., patients with genetic abnormalities affecting epigenetic regulation) [38]. A preliminary report from the randomized DECIDER study suggests that ATRA improves survival of AML patients receiving hypomethylating agents [39], and recent experimental studies also suggest that ATRA induces degradation of the mutated NPM1 protein [40], sensitizes AML cells to FLT3 inhibitors [41], and is effective in AML cells carrying the metabolism-modulating IDH mutations [42]. Second, clinical studies suggest that valproic acid improves survival in certain AML subsets (i.e., patients with NPM1 mutations) [43], and recent experimental studies suggest that it has synergistic effects when combined with autophagy-inhibiting chloroquine [44] or inhibitors of DNA methyltransferase [45]. HDAC-induced epigenetic reprogramming may also increase the susceptibility to ATRA [46]. For future studies, predictive molecular networks may become important to identify responders to valproic acid [47].

Metabolomics is emerging as a powerful approach to study cancer metabolism, and a metabolomics study showed that systemic metabolic markers (i.e., an altered glucose metabolism signature) was associated with prognosis in AML [28]. Such metabolomic signatures probably reflect both the characteristics of the AML cells/disease and the influence of a severe disease on systemic metabolic regulation. The overall systemic metabolomic profiles may also be important for leukemic growth and chemosensitivity. AML cells show metabolic plasticity; however, leukemia growth and chemosensitivity also seem to depend on the environmental context, possibly including the systemic supply of nutrients and especially amino acid and fatty acid metabolism [48,49]. The systemic metabolomic effects of ATRA and valproic acid may thus further contribute to the effects of these agents on AML cells. In this context, we have performed systemic metabolomic profiling of AML patients treated with the combination of ATRA, valproic acid, and low doses of conventional cytotoxic drugs, and we also examined the effects of both ATRA and valproic acid monotherapy on systemic metabolite profiles of AML patients. In our present study, we have focused on metabolite pathways rather than single metabolites. The aims of our study were to compare the pretreatment metabolomic profiles found in responders and non-responders to the AML-stabilizing treatment, and to characterize how these profiles are altered during the early period of this antileukemic treatment (two-day or seven-day monotherapy with either ATRA or valproic acid, respectively).

## 2. Materials and Methods

### 2.1. Patients

The study was approved by the Regional Ethics Committee (REK 2017/305 070417, REK Vest 2015/1410 190615, 215.03 120504, 231.06 150307) and included 44 elderly or unfit patients (25 men and 19 women; median age 76.4 years with range 61–86 years) with high-risk leukemia, i.e., AML relapse, secondary AML, complex karyotype and/or TP53 mutations (Table S1). Patients with APL were excluded. The patients represent an unselected subset of the consecutive patients included in two clinical studies [19,27]. Eighteen of the patients were classified as responders to the treatment according to the myelodysplastic syndrome (MDS) criteria [50]; the others 26 patients did not respond to the treatment and six of them showed a rapidly progressive disease (patients 39–44).

### 2.2. The Antileukemic Treatment Based on ATRA, Valproic Acid, and Low-Dose Cytotoxic Drugs

All serum samples were collected from patients included in two previously published clinical studies conducted at Haukeland University Hospital in Bergen, based on combined treatment with oral ATRA (22.5 mg/m^2^ twice daily for 14 days every 12th week), continuous valproic acid therapy at maximal tolerated doses, and low-dose therapy with hydroxyurea, 5-mercaptopurine, and/or cytarabine [19,27]. Samples were taken prior to treatment, and also after receiving ATRA alone (after two days of treatment) or valproic acid alone (after seven days of treatment), as described in the following sections (see Figure S1).

Patients included in the first study [27] received oral ATRA as described above. On day three of the first cycle the patients received valproic acid as an initial intravenous infusion with a loading dose (5 mg/kg) followed by continuous infusion (28 mg/kg/24 h), and theophylline with a loading dose (5 mg/kg) followed by continuous infusion (0.65 mg/kg/hour). After five days (day 8), the patients received oral valproic acid (serum levels between 300–600 μmol/L) and oral theophylline adjusted to reach the therapeutic level of 50–100 μM. Patients with (i) peripheral blood blasts exceeding 50 × 10^9^/L at diagnosis or (ii) later increasing blast counts received cytotoxic drugs to control leukocytosis.

Patients included in the second study [19] received valproic acid monotherapy for the first seven days with loading dose and subsequent continuous infusion for 24 h before oral valproic acid treatment (serum levels 300–600 μmol/L). On days 8–22, the patients received ATRA, and on days 15–24, they received subcutaneous cytarabine (10 mg/m^2^ once daily). ATRA and cytarabine treatment were repeated at 12-week intervals. Patients with later increasing peripheral blood blasts (>50 × 10^9^/L) received hydroxyurea or 5-mercaptopurine instead of cytarabine.

There is no general agreement with regard to which response criteria should be used for patients receiving low-toxicity AML stabilizing treatment [20], and our patients were therefore evaluated both with regard to the conventional AML criteria for remission induction [51] and the MDS response criteria [52]. Two responders in the second study achieved complete hematological remission; the other responders fulfilled the criteria for hematological improvement according to the MDS criteria and lasting for at least eight weeks before progression.

### 2.3. Preparation and Analysis of Serum Samples

Venous peripheral blood was collected onto sterile serum-clot activator tubes with gel separator (Greiner Bio-One GmbH, Kremsmünster, Austria or BD Vacutainer SST, Becton-Dickenson; Franklin Lakes, NJ, USA). Samples were left upright for 30–120 min at room temperature before being centrifuged (1300× *g,* 10 min). The serum supernatants were immediately allocated in plastic cryotubes (Nunc^TM,^ Roskilde, Denmark) and stored at –80 °C. The patients had no dietary restrictions, and all samples were collected from fasting patients, between 7:30 and 8:30 in the morning before breakfast.

The untargeted metabolomic profile analyses were performed in collaboration with Metabolon Inc (Durham, NC, USA) as described previously [53]. Samples were prepared using the automated MicroLab STAR^®®^ system (Hamilton Company, Bonaduz, Switzerland) and extracted using Metabolon‘s standard solvent extraction method [53]. Four recovery standards were added prior to the first extraction step, DL-2-fluorophenylglycine, tridecanoic acid, d6-cholesterol and 4-chlorophenylalanine [53]. To remove proteins, dissociate small molecules bound to proteins or trapped in the protein matrix, and to recover chemically diverse metabolites, proteins were precipitated with methanol under vigorous shaking for two minutes (Glen Mills GenoGrinder 2000) followed by centrifugation. The resulting extract was divided into two fractions for analysis by two separate reverse phase (RP)/ultra-performance liquid chromatography tandem mass spectrometry (UPLC-MS/MS, Waters ACQUITY, Milford, MA, USA) methods with positive ion mode electrospray ionization (ESI, Thermo Scientific, Waltham, MA, USA) RP/UPLC-MS/MS with negative ion mode ESI, and hydrophilic interaction liquid chromatography (HILIC)/UPLC-MS with negative ion mode ESI. A last sample was stored for backup. Samples were placed briefly on a Zymark TurboVap^®®^ (McKinley Scientific, Sparta, NJ, USA) to remove the organic solvent; they were thereafter stored overnight under nitrogen before further preparation. Extracted water samples served as process blanks and solvents used during the extraction process served as solvent blanks. A pooled matrix sample generated by taking a small volume of each experimental sample, and also a large pool of human plasma extensively characterized and prepared by Metabolon, served as technical replicates throughout the data set. Furthermore, a combination of quality control standards that were carefully chosen not to interfere with the analyzes, were spiked into every analyzed sample to allow monitoring of instrument performance and chromatographic alignment as described previously [53].

Instrument variability was determined for each sample by calculating the median relative standard deviation (RSD) prior to injection into the mass spectrometers. The overall process variability was determined by calculating the median RSD for all endogenous metabolites (i.e., non-instrument standards) present in 100% of the pooled matrix samples (serum samples). The experimental samples were randomly spaced across the platform and the control samples were evenly spread.

Metabolon Inc maintains a library based on authentical standards that contains the retention time/index (RI), mass to charge ratio (*m*/*z*), and chromatographic data (including MS/MS spectral data); the metabolites were identified by comparison to this library. Peaks were quantified using area-under the-curve.

### 2.4. Statistical Analyses

Welch′s two sample *t*-test was used to identify metabolites that differed significantly between different groups, the paired *t*-test was used comparison of paired samples, and ANOVA contrasts used to analyze pharmacological effects for responders and non-responders separately. Random Forest analysis was used to provide an unbiased estimate of how well individuals can be classified into each group based on the metabolomic data. Metabolite pathway enrichment analysis was used for biological interpretation of metabolite data at a system level using the MetaboLync analysis tool (MetaboLync^®®^ Portal). Differences were regarded as statistically significant when *p*-values were < 0.05. Statistical analyses were performed with the programs ArrayStudio, R (http://cran.r-project.org/) and the data analyze software program JMP (JMP^®®^, Statistical Discovery^TM^ from ©SAS Institute Inc., Lane Cove, NSW, Australia).

## 3. Results

### 3.1. The Pretherapy Serum Metabolomic Profiles Could Not Predict Responsiveness to Antileukemic Treatment Based on ATRA, Valproic Acid, and Low-Dose Chemotherapy

The serum metabolomic profiles were analyzed in pretreatment serum samples derived from 44 patients (18 responders and 26 non-responders). Untargeted metabolomic profiling identified 886 metabolites, of which approximately 45 significant changes would be expected to occur by chance alone when using *p* < 0.05 as significance level; however, only 36 metabolites showed a significant difference (*p* < 0.05, Welch two-sample *t*-test) when comparing pretreatment levels of responders and non-responders, and all of these metabolites had high *q*-values (*q* > 0.4). These results suggest that some of the statistically significant changes represent false discoveries (Figure S2). First, 10 of the 36 significantly altered metabolites are classified as peptides (gamma-glutamyltyrosine), amino acids (tyrosine, methionine, histidine, lysine, threonine, homoarginine), or amino acid metabolites (indolepropionate, 2-methylbutyrylcarnitine, 5-hydroxylysinepropionyl) (Figure S2). Second, 11 of the 36 significantly altered metabolites were classified as lipid metabolites (Figure S2). Thirdly, there were also significantly altered levels of five metabolites classified as xenobiotics between responders and non-responders (i.e., metabolites not naturally produced by the organism) and two of these were vitamin A metabolites. Finally, only two metabolites from the subclass carbohydrate/energy metabolism (mannose, citrate) differed significantly when comparing responders and non-responders, and both showed only borderline significance (0.04 > *p >* 0.05).

As stated above, the significantly altered metabolites between responders and non-responders showed high *q*-values in our analysis (Figure S2). Although both fatty acid and amino acid metabolism seem important for the growth and chemosensitivity in human AML [48], and a distinct pretherapy systemic glucose metabolism signature is associated with prognosis (i.e., survival) of younger patients receiving intensive and potentially curative AML therapy [28], we could not identify any highly significant differences in pretreatment systemic metabolite profiles between responder and non-responder patients receiving low-intensity AML-stabilizing treatment. Finally, a random forest analysis based on the overall metabolite profiles showed a predictive accuracy of only 48% for responders versus nonresponders (data not shown).

We also did a metabolite pathway enrichment analysis based on the 36 metabolites with *p* < 0.05 (Figure S3). This analysis showed enriched pathways associated with amino acid and lipid metabolism, although vitamin A metabolism also had a high ranking (two metabolites differed between groups). Taken together, these analyses suggest that, in contrast to patients receiving intensive and potentially curative antileukemic treatment, there is no (or only a minor) prognostic impact of the pretherapy metabolomic profiles in our study for patients receiving leukemia-stabilizing treatment. Although vitamin A metabolism was enriched in the pathway analysis this should be interpreted with great care because only two of the five identified metabolites had borderline significance between responder and non-responder groups (see Figure S2).

### 3.2. Pretherapy Differences in Fatty Acid Metabolism are Found between Non-Responders with Rapidly Progressive AML Compared with Other Non-Responders with Less Aggressive Disease

**Figure 1:**
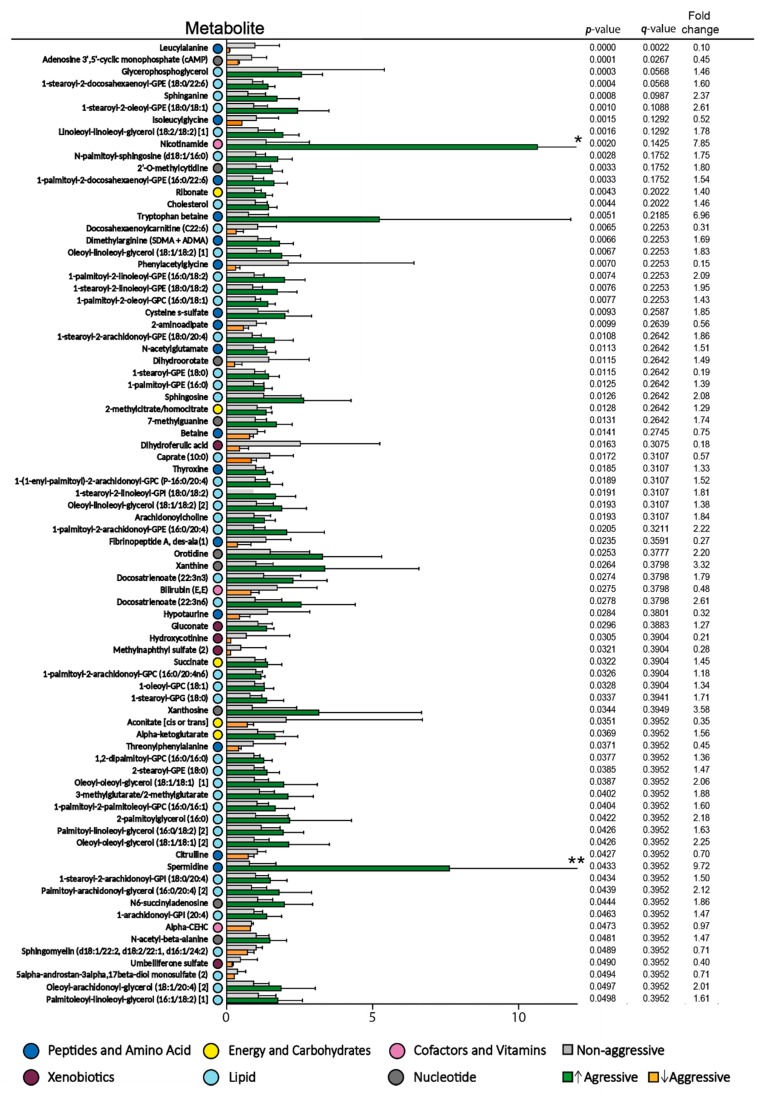
Significantly altered metabolites when comparing the pretreatment levels of non-responders with rapidly progressive disease versus non-responders with less aggressive disease to antileukemic treatment based on all-trans retinoic acid (ATRA) and valproic acid. The *p*-values, *q*-values and mean fold change values for each metabolite are listed to the right in the figure (ranked by *p*-value), and a fold change >1 indicates that the levels were increased in non-responder patients with very aggressive disease compared to non-responder patients with less aggressive disease. Levels of serum metabolites in non-responders with less aggressive disease are shown in grey, increased levels found in non-responders with aggressive disease are shown in green, while decreased levels in non-responders with aggressive disease are shown in yellow. Color codes for classification of metabolites are explained at the bottom of the figure. Error bars show Standard deviation (SD). * Nicotidamine SD 6.761, ** Spermidine SD 9.051.

**Figure 2:**
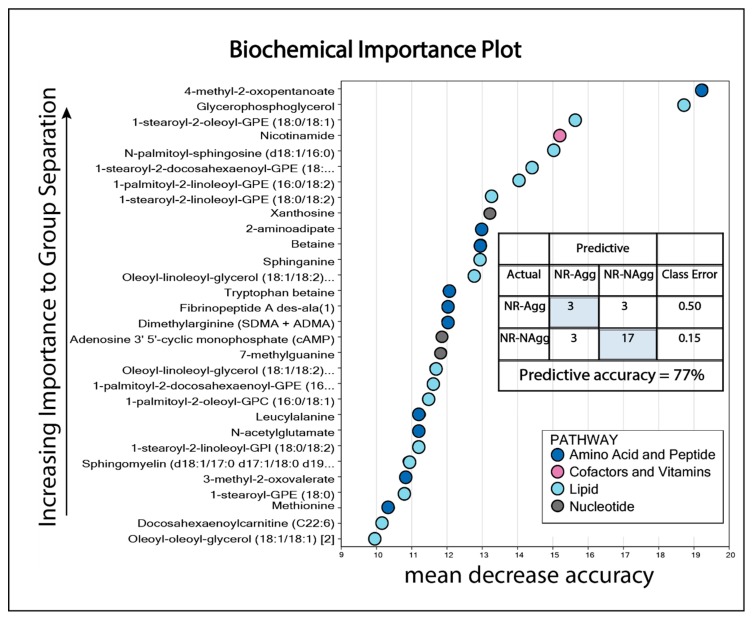
Random forest analysis based on pretreatment serum metabolites when comparing non-responders with rapidly progressive disease versus non-responders with less aggressive disease to antileukemic treatment based on ATRA and valproic acid. The figure shows the 30 top-ranked metabolites from this analysis, which can segregate the two patient groups with a predictive accuracy of 77%. Amino acid and lipid metabolites constituted the majority of the top-ranked metabolites. Color codes for classification of metabolites are shown to the lower right. This analysis included 26 metabolites with a *p*-value < 0.05 (see Figure 1; six amino acid metabolites, 16 lipid metabolites, and four other metabolites). Abbreviations; NR-Agg, non-responders aggressive disease (i.e., rapidly progressive); NR-NAgg, non-responders non-aggressive disease.

Six non-responders had rapidly progressive disease with increasing peripheral blood blast counts and survival ≤12 days (Table S1, patients 39–44). We compared the metabolomic profile of these six patients with the 20 other non-responders because, in clinical practice, it will be important to identify such patients and start early with alternative therapy. This early cutoff was chosen because a clinically relevant response is usually seen after 2–3 weeks of treatment with ATRA/valproic acid [19,27]. Eighty of the 886 metabolites differed significantly between these two groups (Figure 1, Table S2). Most of the metabolites had a relatively high *q*-value, but when considering the relatively high number of significantly altered metabolites (80 out of 886 metabolites) and the overall metabolomic profiles/pathways (instead of individual metabolites), the most striking difference between these two groups was the high number of lipid metabolites (45 out of 80 metabolites) of which patients with rapidly progressive disease showed increased levels for 42 out of these 45 lipid metabolites (a more detailed classification of metabolites is presented in Table S2). Thus, the six non-responders with rapidly progressive disease seem to differ from the 20 other non-responders especially with regard to lipid and fatty acid metabolites. Finally, a random forest analysis showed a predictive accuracy of 77% (Figure 2), suggesting that it might be possible to use metabolite profiles to segregate these groups; however, the group size of aggressive non-responders is very small, and the results therefore need to be confirmed in a larger study. The 30 top-ranked metabolites from the random forest analysis included 26 metabolites with a *p*-value < 0.05 (six amino acid metabolites, 16 lipid metabolites, and four other metabolites; see Figure 1 and Figure 2).

**Figure 3:**
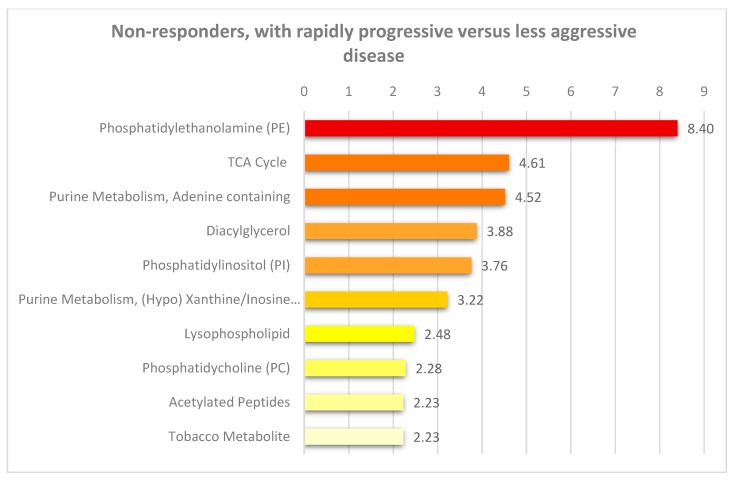
Pathway enrichment analysis based on altered metabolites between non-responder patients with rapidly progressive disease (i.e., aggressive disease) versus non-responders with less aggressive disease. This analysis was based on significantly altered metabolites *p* < 0.05 (see Figure 1). Only pathways with an enrichment value greater than two and at least two metabolites within each pathway are shown in the figure.

We did a pathway enrichment analysis (Figure 3) based on metabolites with *p*-value below 0.05, and mostly fatty acid pathways were enriched between non-responders with rapidly progressive AML and non-responders with less aggressive disease. The metabolites important for the tricarboxylic acid cycle (five metabolites, see Figure 1) all reflect transition of fatty acid metabolites to the energy metabolism/mitochondria. Thus, our results suggest that rapidly progressive AML is associated with alterations especially in lipid (i.e., fatty acid) metabolism.

### 3.3. Effects of ATRA Monotherapy on Serum Metabolite Profiles

**Figure 4:**
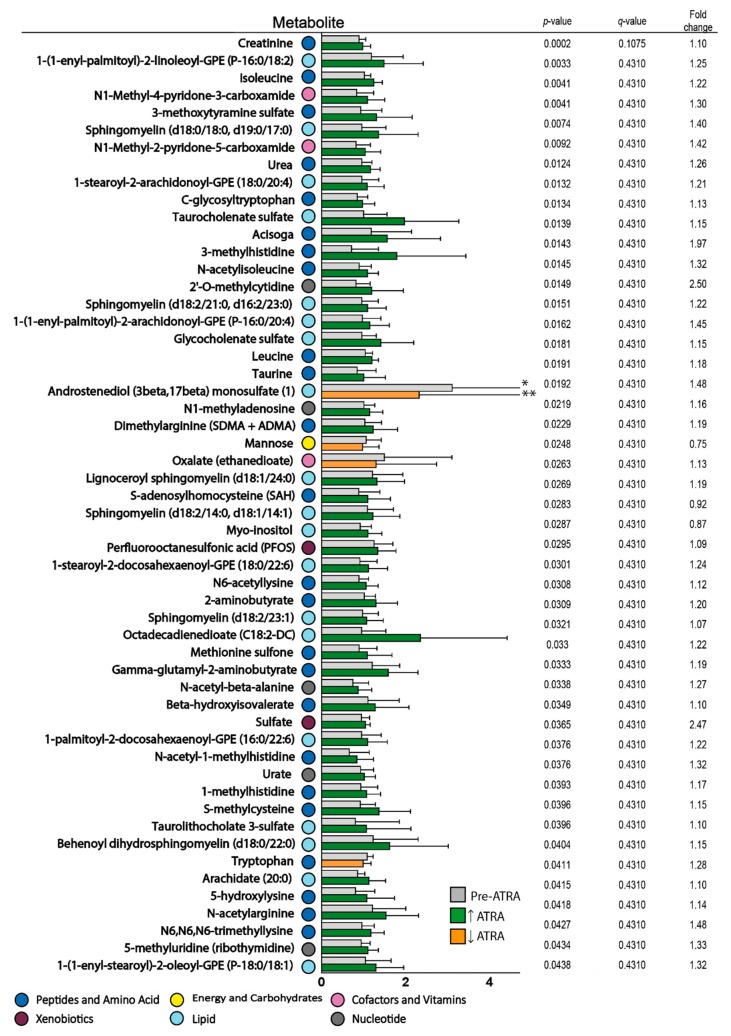
The effect of ATRA monotherapy on the serum metabolomic profiles of patients after two days of treatment. Fifty-four metabolites were significantly altered after ATRA treatment (*p <* 0.05). The *p*-values, *q*-values, and mean fold change values for each metabolite are listed to the right in the figure (ranked by *p*-value), and a fold change >1 indicates that the levels were increased in responders compared with non-responders. Pretherapy levels of serum metabolites for the ten patients are presented in grey, increased levels during ATRA treatment are presented in green and decreased levels presented in yellow. Color codes for classification of metabolites are explained at the bottom of the figure. Error bars show Standard deviation (SD). * Androstenediol (3 beta, 17 beta) pre-ATRA SD 3.862, ** Androstenediol (3 beta,17 beta) post-ATRA SD 2.542.

We compared the serum metabolite profiles before and after two days of ATRA monotherapy for ten patients from the first clinical study (Table S1, the first five responders and five non-responders) [27]. A total of 54 metabolites were then significantly altered after ATRA monotherapy (paired *t*-test, *p* < 0.05, Figure 4), though all metabolites had high *q*-values (*q* > 0.1, Figure 4). However, of the 54 altered metabolites, a large majority of 50 metabolites were increased after ATRA treatment.

A large fraction of these 54 metabolites were amino acid metabolites (24/54), including isoleucine/leucine and tryptophan together with several of their metabolites as well as histidine, lysine, methionine/cysteine/taurine arginine, lysine, and valine metabolites (Figure 4). Nineteen lipid metabolites were also altered by ATRA; especially sphingolipid/sphingomyelin but also plasmalogen metabolites were increased. The largest subgroup of altered lipid metabolites was sphingolipids that are largely synthesized from serine and palmitoyl-CoA and are important as cell membrane components and regulators of cell proliferation and survival [54,55,56]. Although several sphingomyelins were increased, their second messenger ceramide was not altered. Finally, several one-carbon/methylated metabolites were increased after ATRA therapy together with the methylation reaction product S-adenosylhomocysteine.

We performed a pathway enrichment analysis (Figure S4) based on the significantly altered metabolites observed after ATRA treatment. This analysis also showed that especially lipid metabolites but also amino acid metabolites before and during ATRA therapy. In addition, we finally also investigated the effects of ATRA monotherapy for the five responders and the five non-responders separately. A similar profile was then observed where significantly altered metabolites (*p* < 0.05, but *q* > 0.05) consisted mainly of amino acid and lipid metabolites in both groups (data not shown).

To conclude, our present metabolomic results suggest that ATRA mainly increases levels of metabolites involved in amino acid and lipid metabolism, but these observations must be interpreted with great care because the effects were relatively weak and showed high *q*-values. However, previous animal studies have also shown that ATRA can alter the systemic levels of amino acid and fatty acid metabolites [37]; these results are thus consistent with our present observations in AML patients.

### 3.4. Valproic Acid Monotherapy Alters the Systemic Amino Acid and Lipid Metabolite Profiles in Both Responders and Non-Responders to the Antileukemic Treatment

**Figure 5:**
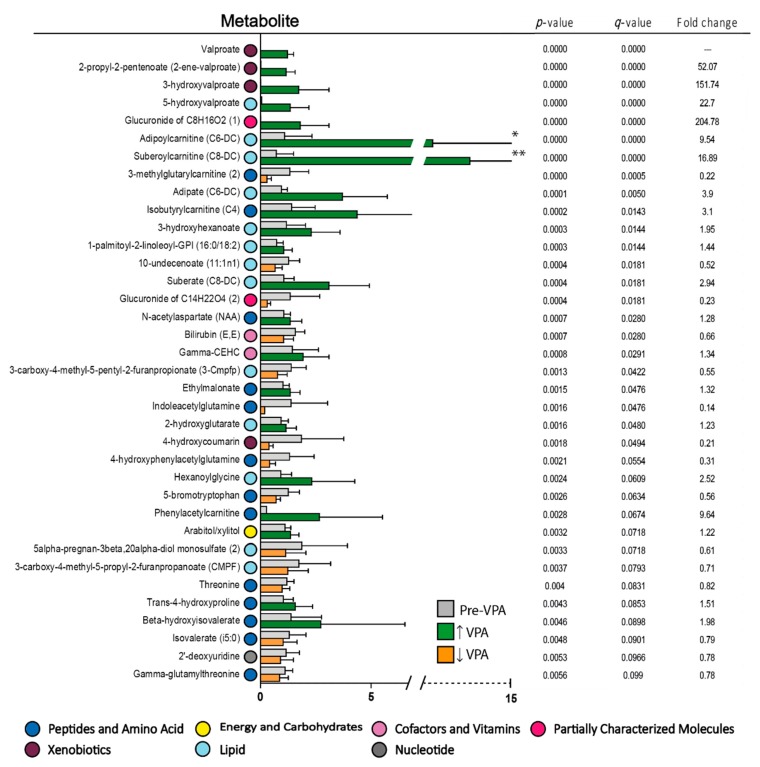
Identification and classification of serum metabolites that differed significantly when comparing samples taken prior to treatment and after seven days of valproic acid (VPA) therapy. Thirty-six metabolites differed significantly between untreated and VPA-treated samples (*p* < 0.05, Welch′s two sample *t*-test), with *q*-value < 0.1 (the upper 23 metabolites with *q* < 0.05). The *p*-values, *q*-values, and mean fold change for each metabolite are listed to the right in the figure (ranked by *p* and *q*-value), and a fold change >1 indicates that the levels were increased after valproic acid therapy. Metabolite levels found in pretreatment samples are shown in grey, while increased levels during treatment are shown in green (22/36 increased) and decreased levels during treatment are shown in orange (14/36 decreased). Color codes for classification of metabolites are explained at the bottom of the figure. Error bars show Standard deviation (SD). *Adipoylcranitine (C6-DC) SD 6.928 **Suberoylcarnitine (C8-DC) SD 8.50.

We compared the systemic metabolomic profiles before and after seven days of valproic acid monotherapy for ten patients included in the second clinical study, including the first five responders and non-responders (Table S1) [19]. A total of 109 metabolites were significantly altered in patients treated with valproic acid compared to pretreatment levels; 55 metabolites were increased, and 54 were decreased (*p* < 0.05; see Figure S5). Among these, 36 metabolites had both a *p*-value < 0.05 and a *q*-value < 0.1 (Figure 5), and 23 of these metabolites also had a *q*-value below 0.05. Four of these 36 metabolites were valproic acid metabolites, whereas the rest included 13 amino acid metabolites and 12 lipid metabolites. Only one of these metabolites reflected energy/carbohydrate metabolism.

**Figure 6:**
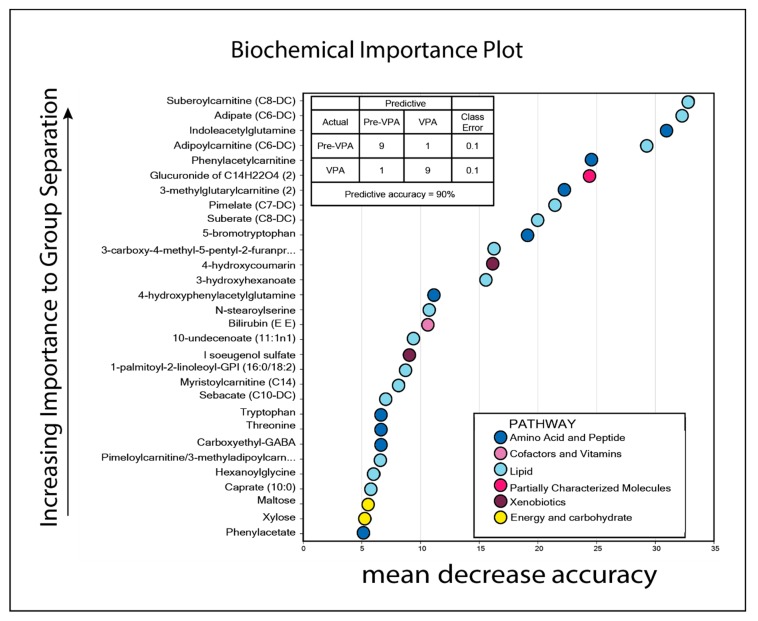
The effect of 7-day valproic acid (VPA) monotherapy on the serum metabolomic profiles for ten patients (five responders and five non-responders). The random forest analysis was based on the identification of 886 metabolites in pretherapy samples and samples collected after seven days of valproic acid monotherapy. The analysis showed a predictive accuracy of 90% (see the insert table) after exclusion of the four valproic acid metabolites. The top-30 most important metabolites for separation of the two groups are shown in ranking order. Color codes indicate the classification of individual metabolites at the lower right part of the figure.

A random forest analysis was performed after excluding the four valproic acid metabolites, and then 14 lipid and seven amino acid metabolites were among the 30 top-ranked metabolites of this analysis, with a predictive accuracy of 90% (Figure 6). Twenty-two of these 30 metabolites were significantly altered by the valproic acid treatment (*p* < 0.05, see Figure S5), including six of the highly significant carnitine metabolites (Figure 6). In addition, another random forest analysis was performed including all identified metabolites (not excluding valproic acid metabolites) and the results showed that the four top-ranked metabolites were all valproic acid metabolites as expected (Figure S6; predictive accuracy of 100%). Hence, responsiveness to the combination of ATRA and valproic acid is probably not determined by a difference in valproic acid metabolism.

**Figure 7:**
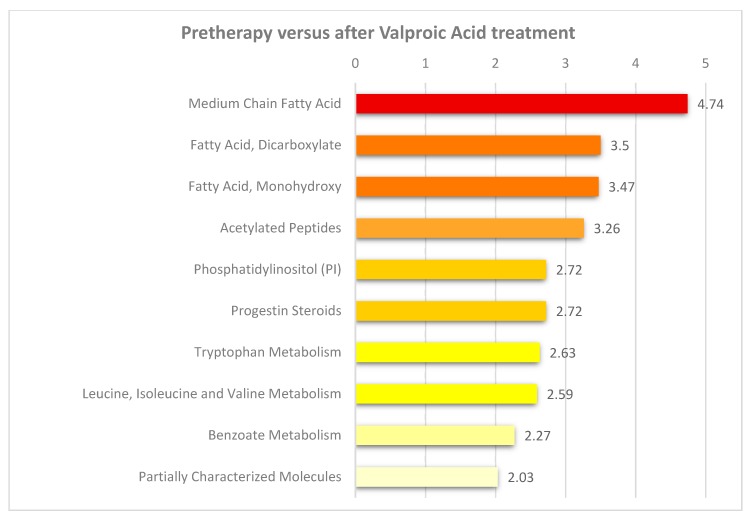
Pathway enrichment analysis based on metabolites altered after seven days of valproic acid treatment compared to pretherapy levels. This analysis was based on significant altered metabolites *p* < 0.05 (see Figure S5), and only pathways with an enrichment value greater than two and at least two metabolites within each pathway are shown. The most significant pathway is shown in red and less significant pathways in light yellow.

A pathway enrichment analysis for differential metabolites after valproic acid treatment revealed enrichment of pathways relating especially to lipid metabolism as well as amino acid metabolism (Figure 7). As discussed above, several lipid and fatty acid metabolites were altered after valproic acid therapy, including increased levels of several fatty acid-carnitines (Figure 5, Figure S5). This is consistent with impaired β-oxidation, and the increased levels of five out of nine significantly altered dicarboxyl fatty acids suggests that ω-oxidation is used as an alternative mechanism in the liver to compensate for impaired β oxidation [57]. Furthermore, a total of 33 amino acid metabolites were significantly altered by valproic acid, in particular metabolites reflecting tryptophan (six metabolites) and valine/leucine/isoleucine metabolism (eight metabolites). Seven amino acid metabolites were also included among the top-ranked metabolites in the random forest analysis (Figure 6). In our analysis, the 109 metabolites with significant *p*-values < 0.05 (but high *q*-value) had a similar overall profile as that found for metabolites with *p* < 0.05 and *q* < 0.05 (Figure 5), mainly consisting of metabolites belonging to amino acid (see Figure S7) and lipid pathways (see Figure S8), whereas metabolites reflecting the carbohydrate and energy metabolism were scarce.

We finally analyzed and compared the effects of valproic acid monotherapy for the five patients classified as responders and five non-responders separately (Table 1). Then, 78 metabolites were significantly altered after valproic acid treatment for the responders, including divergent effects for 17 amino acid and 32 lipid metabolites, while 105 metabolites were significantly altered after valproic acid treatment for the non-responders, including divergent effects for 21 amino acid and 49 lipid metabolites (Table 1, Tables S3 and S4). Thus, a similar pattern was observed for both responders and non-responders as described above; valproic acid mainly alters amino acid and lipid metabolism. The metabolites showing both *p*- and *q*-value < 0.05 are listed in Table 1. These metabolites included the valproic acid metabolites and also several carnitines, which also reflect differences in lipid and amino acid metabolism.

## 4. Discussion

Epigenetic targeting based on combination therapy with ATRA and valproic acid can stabilize the disease for a subset of AML patients. Furthermore, both drugs can also modulate the cellular and/or systemic metabolism [32,33,34,35,36,37]. Such pharmacological effects may influence the metabolic regulation of the leukemia cells through altered levels of nutrients in their microenvironment [48,49], and these effects may even contribute to the epigenetic effects of the drugs [58,59]. We have therefore performed metabolomic profiling of serum samples collected from AML patients before receiving disease-stabilizing treatment to compare responders and non-responders to treatment, and we also compared systemic metabolite profiles before and after drug monotherapy. Our study suggests that differences in pretreatment amino acid and lipid metabolite profiles are associated with disease aggressiveness, and both ATRA and valproic acid cause further modulation of the systemic metabolic signature (i.e., amino acid and lipid metabolism). This is different from patients receiving intensive and potentially curative chemotherapy where a glucose metabolism signature has been shown to be associated with chemoresistance and survival after chemotherapy [28].

Untargeted metabolomics generates large amounts of data that can be complex and challenging to analyze. A *p*-value of 0.05 is the false positive rate when one test is performed, but for a large number of tests on the data one has to account for false positives. There are different methods to correct for multiple testing. One strategy is to use the family-wise error rate adjustment (e.g., Bonferroni correction), but this is regarded as very conservative when one has a large number of tests; the use of false discovery rates (i.e., the *q*-values) is therefore more common although it allows for a small number of false discoveries [60]. A relatively high *q*-value (e.g., *q* > 0.10) indicates diminished confidence but it does not necessarily rule out the biological significance of a result. In our opinion other evidence should also be considered, for example (i) significance in another dimension of the study, (ii) inclusion in a common pathway together with highly significant compounds, and (iii) residing in a similar metabolic family with other statistically significant metabolites. Throughout the present study we have therefore for each biological comparison listed all metabolites with the corresponding *p*-value < 0.05, we have presented *p*-values together with the corresponding *q*-values and fold change values for all these metabolites, and when we discuss the importance of single metabolites we focus on the *q*-values (i.e., the studies of the valproic acid effects). We also performed pathway enrichment analysis based on significantly altered metabolites (*p* < 0.05) which revealed enriched pathways instead of focusing on single metabolites. In our opinion such an identification of a metabolic pathway can be justified because it is based on several metabolites and not a single metabolite. However, we would like to emphasize that all background information (*p*- and *q*-values and fold change values) are provided in the text or in figures/tables for all metabolites included in our analyses, so the level of confidence in the results can be further evaluated by the reader.

Our pretreatment profiles were probably determined by the metabolic characteristics of a large leukemia cell burden together with the effects of this burden on systemic metabolic regulation. The lipid and amino acid profiles were further modulated by metabolic effects of ATRA but especially of valproic acid. The drugs have direct (i.e., epigenetic) effects on the AML cells, and metabolic modulation may then represent an additional indirect effect on the leukemia cells by modulation of metabolite/nutrient levels in their bone marrow microenvironment. Thus, altered metabolomic profiles may contribute for the prognostic evaluation for such patients, but they may also influence AML cell chemosensitivity and/or contribute to the antileukemic effects of the treatment.

The clinically relevant responses to this AML-stabilizing treatment are usually seen after 14–20 days of treatment [19,27]. Thus, in this study, the effects of ATRA and valproic acid on metabolomic profiles were evaluated early before improvement of peripheral blood cell counts could be expected. Despite the late clinical responsiveness, alterations in metabolomic signatures were found during the first days of treatment and these effects differed between patients whom about two weeks later became responders or non-responders to treatment.

There is no general agreement on how responses to AML-stabilizing treatment should be classified. We used the generally accepted definition of complete hematological remission for AML patients [51] and the MDS criteria for stable disease that require improvement/stabilization of normal peripheral blood cell counts for at least two months [61]. However, the median survival for elderly patients not receiving AML-directed therapy is only two-three months [7,62,63] and we therefore regarded such disease stabilization as unexpected and classified this as a response.

Amino acid profiles in healthy individuals and cancer patients have been compared in several previous studies [64]. The plasma amino acid profiles for patients with non-small-cell lung cancer or colorectal cancer is considerably different from healthy controls, and altered amino acid profiles have been detected also in early stage breast cancer [64]. Our present study found altered amino acid profiles in AML after both ATRA and valproic acid treatment, and we observed a considerable heterogeneity in pretherapy metabolite levels among AML patients when comparing (i) responders versus non-responders and (ii) non-responders with rapidly progressive disease versus other non-responders. The mechanisms behind altered amino acid profiles in cancer patients may be related to the risk of cancer [65] or reflect nutritional or microbiotic alterations, progression of the disease, cachexia or weight loss [66,67,68]. In our opinion, it is most likely that the mechanism behind the differences in amino acid metabolism between various AML patient groups is also multifactorial.

To the best of our knowledge, the present study is the first to investigate metabolomic profiles in patients receiving AML-stabilizing treatment. We investigated elderly and unfit patients that were included in two previous clinical studies; in both clinical studies the patients received the same doses of ATRA, guidelines for adjustment of valproic acid treatment were similar, and we used low-dose (i.e., low-toxicity) cytarabine/mercaptopurin/hydroxyurea. Our results showed that responders and non-responders showed relatively small differences in pretherapy metabolic profiles, but non-responders with rapidly progressive disease seemed to have more extensive differences compared with the other non-responders. Fatty acid oxidation may be important for AML growth [69], and increased levels of lipids/fatty acids in non-responders could then be a strategy to provide energy substrates for the growing leukemia cells. In addition, the long chain fatty acid oleoyl ethanolamid is suggested to contribute to drug resistance in other patients with hematological malignancies [70]. The increased oleoyl ethanolamid levels may also reflect cachexia that is associated with poor prognosis in other malignancies [68]. Our responders and non-responders differed in their pretherapy levels of the three endocannabinoids oleoyl ethanolamide, linoleoyl ethanolamide (both increased in non-responders) and *N*-palmitoylserine (decreased in non-responders). Oleoyl ethanolamide activates the nuclear peroxisome proliferator-activated receptor alpha (PPAR-alpha) that induces lipolysis and fat utilization [71]. Consistent with increased lipolysis and energy utilization, non-responders also showed significantly altered serum levels of four free fatty acids and one fatty acid metabolite, suggesting release of fatty acids. Oleoyl ethanolamide may therefore represent a link between endocannabinoid and fatty acid metabolism in different patient groups. In addition, responders and non-responders also differed in xenobiotic metabolites including gut-bacteria derived metabolites. Taken together, these observations suggest that differences in nutritional status or gut microbiome contribute to the metabolomic heterogeneity of elderly patients with newly diagnosed AML.

Our analyses showed that the non-responders are not only heterogeneous with regard to survival but also with regard to pretherapy metabolomic profiles. Patients with rapidly progressive disease differed considerably from other non-responders especially in lipid metabolism but also amino acid metabolism. AML is a very aggressive disease and if the first treatment fails the patients may become unfit for further treatment. Our present observations have to be interpreted with great care because few patients with very aggressive disease were studied and further studies are needed. Still, they suggest that pretreatment metabolomic profiling may be helpful to select patients that should not receive treatment based on ATRA plus valproic acid, especially patients that have rapidly progressive disease because it takes 15–20 days before clinical responses can be detected.

Neither our comparisons of pretherapy metabolomic profiles of responders and non-responders nor our studies of metabolite profiles during monotherapy detected any major differences in carbohydrate or energy metabolism (glycolysis, citric acid cycle), even though such differences have a prognostic impact for AML patients receiving intensive therapy [28]. However, clinically relevant responses to our disease-stabilizing therapy can be seen even for patients with high-risk AML (e.g., high-risk karyotype, relapse; Table S1). Thus, the conventional high-risk criteria, and also glucose metabolism, seem less important for patients receiving treatment based on ATRA and valproic acid. Instead, differences in or altered lipid and amino acid metabolism seem to be important for patients receiving this disease-stabilizing treatment, and effects on systemic levels of metabolites induced by valproic acid generally seem to be stronger than the ATRA-induced effects. Previous studies have also described an effect of ATRA on one-carbon metabolism [72], and similar effects were also seen in our AML patients although they seemed to be relatively weak.

We also examined the effects of ATRA and valproic acid on metabolomic profiles for ten patients, i.e., five responders and five non-responders analyzed together, after relatively short duration of treatment (two and seven days, respectively); whereas clinically relevant responses are usually detected after two-three weeks [19,27]. Thus, the observed alteration of metabolite profiles should be regarded as early pharmacological effects that are common for responders and non-responders.

The most striking effects by the two drugs in our studies were altered lipid and amino acid metabolism. ATRA increased serum levels of sphingolipids and sphingomyelins in non-responders; a hypothesis is therefore that ATRA contributes to chemoresistance in the non-responders through a growth-enhancing and antiapoptotic effect of these metabolites as described for other malignancies [54,55,56]. Several sphingomyelins were altered by ATRA, but their second messenger ceramide derived from hydrolysis of sphingomyelin was not altered. Altered ceramide synthesis has been observed in other malignancies [73], and a subset of AML patients may even have mutations in the sphingomyelin/ceramide pathway [74]. Taken together, these observations suggest that ATRA also has complex effects on this pathway. Moreover, the levels of several plasmalogens were also altered by ATRA. These glycerophospholipid derivatives are thought to be protective against reactive oxygen species [75], their levels are high in inflammatory cells [76], and their levels increase during differentiation-induction in the HL60 human AML cell line [77]. Thus, increased plasmalogens may reflect ATRA-induced effects on AML cells that contribute to chemoresistance at least for certain patients.

Furthermore, several methylated metabolites were increased after ATRA treatment, including 3-methylhistidine, *N*6,*N*6,*N*6-trimethylhistidine, S-methycysteine, 2′-*O*-methylcytidine, *N*1-methyl-4-pyridone-3-carboxamide, as well as the methylation reaction product S-adenosylhomocysteine. ATRA has been shown to increase both glycine *N*-methyltransferase that regulates the methyl group supply for S-adenosylmethionine-dependent transmethylation reactions [77] as well as the activation of histone methyltransferase SUV39H2 that is important for epigenetic regulation of gene transcription [78]. ATRA-induced alteration of DNA methylation has also been described in the HL60 AML cell line [79]. Thus, these observations suggest that ATRA influences the general methylation potential in AML cells.

Lipid and amino acid metabolism were both altered during valproic acid therapy, and especially altered levels of fatty acid metabolites were observed. There seem to be several links between epigenetic regulation and fatty acid metabolism in AML [58], including expression of fatty acid binding protein 4 (FABP4) in AML cells that is important both for fatty acid uptake and epigenetic regulation [80]. Epigenetic regulation is also important for the expression of acetyl-CoA carboxylase 2, a key driver of fatty acid β-oxidation in AML cells [81] and a regulator of the general lipid metabolism [82]. Repression of this gene by histone deacetylation allows for simultaneous β-oxidation and fatty acid synthesis to take place [81]. Finally, valproic acid has been shown to alter lipid metabolism and fatty acid oxidation [33,35]. Our overall results thereby support the hypothesis that valproic acid alters epigenetic regulation both through direct effects on histone acetylation and indirectly through modulation of fatty acid metabolism.

We compared the effects of ATRA and valproic acid for five responders and five non-responders; these results have to be interpreted with great care due to the low number of samples. However, non-responders showed more extensive effects on amino acid and lipid metabolism after two days of ATRA monotherapy but especially after seven days of valproic acid monotherapy. In our opinion, these more extensive effects in non-responders may partly reflect pharmacological effects, but may also be influenced by disease progression [30,31,32,33,34,35,36].

## 5. Conclusions

Both ATRA and valproic acid have been used in the treatment of AML, and this is the first study to investigate systemic metabolomic profiles in AML patients receiving leukemia-stabilizing treatment based on ATRA plus valproic acid. Our study shows that especially amino acid and lipid metabolism varies between patients and during treatment. The effect of valproic acid on the regulation of lipid and amino acid metabolism is particularly strong and may contribute to the antileukemic and/or epigenetic effects of this drug, whereas the effects of ATRA and the differences between patient subsets are weaker. Metabolites related to carbohydrate or energy metabolism showed only minor variations in our study. Systemic metabolomics should be further investigated to identify biomarkers for pretreatment evaluation of susceptibility to AML stabilizing treatment, though larger studies are needed. Finally, changes in metabolomic profiles may influence the bone marrow microenvironment and thereby modulate AML cell metabolism as well as epigenetic regulation and contribute to therapy resistance.

## Figures and Tables

**Table 1:** An overview of significantly altered serum metabolites (*p* < 0.05 and *q* < 0.05) after seven-day valproic acid therapy.

Biochemical Name	Classification	During VPA Therapy/Pretherapy		
		*p*-Value	*q*-Value	Fold Change
**RESPONDERS**				
*Valproate	Drug concentration	0.0000	0.0000	
*2-propyl-2-pentenoate (2-ene-valproate)	Valproic acid metabolite	0.0000	0.0000	55.81
*5-hydroxyvalproate	Valproic acid metabolite	0.0000	0.0000	13.83
*3-hydroxyvalproate	Valproic acid metabolite	0.0000	0.0000	25.77
*Glucuronide of C8H16O2 (1)*	Partially characterized	0.0000	0.0000	28.34
*Suberoylcarnitine (C8-DC)	Fatty acid metabolism, acyl carnitine	0.0000	0.0000	30.82
Phenylacetylcarnitine	Acetylated peptide	0.0000	0.0000	17.30
*Adipoylcarnitine (C6-DC)	Fatty acid metabolism	0.0000	0.0000	17.06
*3-methylglutarylcarnitine (2)	Leucine/isoleucin/valine metabolism	0.0000	0.0000	0.18
*Adipate (C6-DC)	Fatty acid, dicarboxylate	0.0000	0.0027	3.41
10-undecenoate (11:1n1)	Medium chain fatty acid	0.0001	0.0043	0.49
*Isobutyrylca′rnitine (C4)	Leucine/isoleucin/valine metabolism	0.0001	0.0093	3.06
Glucuronide of C14H22O4 (2)	Partially characterized	0.0003	0.0182	0.22
4-hydroxycinnamate sulfate	Tyrosine metabolism	0.0004	0.0259	3.00
Isoeugenol sulfate	Food component, plant	0.0005	0.0264	0.04
**NON-RESPONDERS**				
*Valproate	Drug concentration	0.0000	0.0000	
*2-propyl-2-pentenoate (2-ene-valproate)	Valproic acic metabolite	0.0000	0.0000	48.32
*5-hydroxyvalproate	Valproic acic metabolite	0.0000	0.0000	31.58
*3-hydroxyvalproate	Valproic acic metabolite	0.0000	0.0000	93.38
*Glucuronide of C8H16O2 (1)	Partially characterized	0.0000	0.0000	16.16
*Suberoylcarnitine (C8-DC)	Fatty acid, dicarboxylate	0.0000	0.0000	12.96
*Adipoylcarnitine (C6-DC)	Fatty acid metabolism	0.0000	0.0000	7.34
*Adipate (C6-DC)	Fatty acid, dicarboxylate	0.0000	0.0001	4.33
*3-methylglutarylcarnitine (2)	Leucine/isoleucin/valine metabolism	0.0000	0.0001	0.25
Suberate (C8-DC)	Fatty acid, dicarboxylate	0.0000	0.0022	3.43
3-carboxy-4-methyl-5-pentyl-2-Furanpropionate (3-Cmpfp)	Fatty acid metabolism, dicarboxylate	0.0001	0.0054	0.44
Hexanoylglycine	Fatty acid metabolism, acyl glycine	0.0001	0.0077	3.03
3-hydroxyhexanoate	Fatty acid metabolism, monohydroxy	0.0001	0.0100	2.03
Androstenediol (3beta,17beta) disulfate (1)	Androgenic steroid	0.0003	0.0197	1.45
*Isobutyrylcarnitine (C4)	Leucine/isoleucin/valine metabolism	0.0004	0.0208	3.15
Gamma-CEHC	Cofactors/vitamins	0.0004	0.0234	1.56
Isoursodeoxycholate	Secondary bile acid metabolism	0.0005	0.0278	0.06
Indoleacetylglutamine	Tryptophane metabolism	0.0006	0.0298	0.09
4-hydroxyphenylacetylglutamine	Acetylated peptide	0.0008	0.0352	0.28
1-palmitoyl-2-linoleoyl-GPI (16:0/18:2)	Phosphatidylinositole	0.0008	0.0352	1.51
5-bromotryptophan	Tryptophane metabolism	0.0010	0.0387	0.49
4-allylphenol sulfate	Food component, plant	0.0010	0.0387	0.29
*N*-acetyltyrosine	Tyrosine metabolism	0.0011	0.0413	2.42
Trans-4-hydroxyproline	Urea cycle. Proline metabolism	0.0011	0.0411	1.73
Galactonate	Carbohydrate metabolism	0.0012	0.0430	0.38
3-aminoisobutyrate	Pyrimidine metabolism, thymine	0.0013	0.0433	1.86

The metabolites are ranked according to their *p*- and *q*-values, and the metabolites that were altered in responders and non-responders to the antileukemic AML-stabilizing therapy are listed separately. Overlapping metabolites between these two groups are marked with *. VPA, valproic acid.

## Supplementary Materials

The following are available online at https://www.mdpi.com/2073-4409/8/10/1229/s1, Figure S1: Timeline of treatment schedule for patients included in two clinical studies, Figure S2: Identification and classification of metabolites in pretherapy serum samples that differed significantly between responders and non-responders to the antileukemic treatment of ATRA plus valproic acid, Figure S3: Comparison of pretreatment metabolomics profiles for responders and non-responders to antileukemic treatment based on ATRA and valproic acid; a metabolite pathway enrichment analysis, Figure S4: Comparison of metabolomics profiles before and during ATRA treatment for responders and non-responders to antileukemic treatment based on ATRA and valproic acid; a metabolite pathway enrichment analysis, Figure S5: The effect of seven-day valproic acid (VPA) monotherapy on the serum metabolomic profiles of AML patients, Figure S6: The effect of valproic acid monotherapy for seven days on the serum metabolomics profiles for 10 patients (five responders and five non-responders; patients included in the study described in PMID 23915396, valproic acid metabolites included in the study), Figure S7: The effect of seven days of valproic acid monotherapy on serum metabolomic profiles; significantly altered amino acid and peptide metabolites when comparing samples derived from 10 patients (five responders and five non-responders; all patients included in the study by Fredly et al. PMID 23915396), Figure S8: The effect of seven days of valproic acid monotherapy on serum metabolomic profiles; significantly altered lipid metabolites when comparing samples derived from 10 patients (five responders and five non-responders; all patients included in the study by Fredly et al. PMID 23915396), Table S1: Clinical and biological characteristics of the included patients, Table S2: Significantly altered serum metabolites between subsets of non-responders to antileukemic treatment based on ATRA and valproic acid; a comparison of non-responders with very aggressive (i.e., rapidly progressive) and less aggressive disease, Table S3: Significantly altered metabolites after seven days of valproic acid monotherapy; a comparison of pretreatment samples versus samples collected during treatment for patients classified as responders to antileukemic therapy based on ATRA and valproic acid, Table S4: Significantly altered metabolites after seven days of valproic acid therapy; a comparison of pretreatment samples versus samples collected during treatment for patients classified as non-responders to antileukemic treatment based on ATRA and valproic acid.
