# Supplementary material for: Systemic Metabolomic Profiling of Acute Myeloid Leukemia Patients before and During Disease-Stabilizing Treatment Based on All-Trans Retinoic Acid, Valproic Acid, and Low-Dose Chemotherapy
Source: Cells. 2019 Oct 10;8(10):1229. doi: 10.3390/cells8101229 (PMC6829623; doi:10.3390/cells8101229)
Supplement: Supplementary file 1 [file cells-08-01229-s001.pdf]

SUPPLEMENTARY INFORMATION

# Systemic Metabolomic Profiling of Acute Myeloid Leukemia Patients before and During Disease-Stabilizing Treatment Based on All-Trans Retinoic Acid, Valproic Acid, and Low-Dose Chemotherapy

**Ida Sofie Grønningsæter [1,2], Hanne Kristin Fredly [3], Bjørn Tore Gjertsen [1,2], Kimberley Joanne Hatfield [2,4] and Øystein Bruserud [1,2] ***

[1] Department of Medicine, Haukeland University Hospital, Bergen 5021, Norway; Ida.Gronningseter@uib.no (I.S.G.); Bjorn.Gjertsen@uib.no (B.T.G.)
[2] Section for Hematology, Institute of Clinical Science, University of Bergen, Bergen 5021, Norway; Kimberley.Hatfield@uib.no
[3] Department of Medicine, Bærum Hospital, Bærum 1346, Norway; Harfre@vestreviken.no
[4] Department of Immunology and Transfusion Medicine, Haukeland University Hospital, Bergen 5021, Norway
* Correspondence: oystein.bruserud@helse-bergen.no; Tel.: +47-55973082; Fax +47-55972950

**<u>Table S1.</u> Clinical and biological characteristics of the included patients[1].** Responders to treatment are listed at the upper part of the table; the non-responders are listed at the lower part.

| ID[1] | Gender | Age | Previous disease | FAB | Membrane molecule expression[2] | | | | | Karyotype | FLT3 | NPM1 | Additional mutations | WBC counts ($x10^8$) | Survival (days)[3] |
|---|---|---|---|---|---|---|---|---|---|---|---|---|---|---|---|
| | | | | | CD13 | CD14 | CD15 | CD33 | CD34 | | | | | | |
| RESPONDERS | | | | | | | | | | | | | | | |
| 1* | M | 73 | MDS | M1 | + | - | - | + | + | multiple | ITD, TKD | wt | PHF6, RUNX1 | <0.5 | 392 |
| 2* | F | 61 | 1st relapse | M1 | + | - | + | + | + | multiple | wt | wt | NRS, SF3B1 | 55.8 | 644 |
| 3* | M | 62 | 2nd relapse | M2 | + | - | + | + | + | -7 | wt | wt | nt | 4.9 | 350 |
| 4* | M | 80 | *de novo* | M1 | + | - | - | + | + | multiple | wt | wt | nt | 8 | 58 |
| 5* | M | 78 | MDS | M1 | + | - | - | + | + | nt | nt | nt | nt | 142 | 69 |
| 6* | F | 68 | 1st relapse | M1 | + | - | + | + | + | normal | wt | wt | TET2, ASXL1, BCOR | 15.6 | 105 |
| 7* | F | 81 | MDS | M1 | + | - | - | + | + | normal | wt | wt | TET2, ASXL1, GATA2, CEBPA, SRSF2 | 6 | 147 |
| 8* | M | 86 | *de novo* | M4 | + | + | + | + | - | nt | nt | nt | nt | 18.7 | 59 |
| 9 | M | 81 | Polycytemia-vera | M2 | - | - | - | - | + | -7 | wt | wt | ASXL1, SRSF2, RAD21 | 22.3 | 610 |
| 10 | M | 77 | MDS | M1 | + | - | - | + | + | normal | wt | wt | NRAS, RUNX1, CEBPA, SRSF1, STAG2 | 2.1 | 419 |
| 11 | F | 85 | MDS | M1 | - | - | - | - | + | multiple | nt | nt | nt | 3.9 | 171 |
| 12 | F | 83 | MDS | M2 | + | - | + | - | + | normal | nt | nt | nt | 0.3 | 196 |
| 13 | M | 66 | MDS | M2 | - | - | + | - | - | 14q | nt | nt | nt | 2,6 | 239 |
| 14 | M | 83 | MDS | M0 | - | - | - | - | + | normal | wt | wt | nt | 1.1 | 102 |
| 15 | M | 73 | *de novo* | M1 | | | | | | nt | wt | Ins | TKD, IDH2, SRSF1 | 12.1 | 383 |
| 16 | M | 74 | *de novo* | M0 | + | - | - | + | + | multiple | wt | wt | TP53 | 18.7 | 151 |
| 17 | F | 72 | MDS | M2 | + | - | - | + | - | t(1;5), t(2;3) | ITD | wt | KMT3, RUNX1 | 42.6 | 132 |
| 18 | F | 77 | MDS | M2 | + | - | - | + | + | normal | wt | wt | NRAS, TET2, ASXL1, RUNX1, SRSF1, STAG2, BCDR | 142 | 132 |
| NON-RESPONDERS | | | | | | | | | | | | | | | |
| 19* | F | 81 | *de novo* | M1 | + | - | - | + | + | multiple | nt | nt | nt | 1.7 | 192 |
| 20* | M | 74 | *de novo* | M0 | + | - | - | - | + | multiple | wt | wt | IDH2 | 13.3 | 112 |
| 21* | F | 70 | MDS | M1 | + | - | - | + | + | multiple | wt | wt | TP53 | 3.6 | 142 |
| 22* | M | 67 | 1st relapse | Nt | nt | nt | nt | nt | nt | normal | TKD | wt | nt | 15.6 | 73 |

| | | | | | | | | | | | | | | | |
|---|---|---|---|---|---|---|---|---|---|---|---|---|---|---|---|
| 23* | M | 71 | 1st relapse | M2 | + | - | - | + | + | normal | TKD | Ins | TET2, GATA2, STAG2 | 1.5 | 49 |
| 24 | F | 82 | *de novo* | M5 | + | - | + | + | + | normal | ITD, TKD | wt | WT1, DNMT3 | 142 | 37 |
| 25 | F | 79 | *de novo* | M2 | + | - | + | + | + | multiple | wt | wt | TP53, BCORL1 | 1.7 | 42 |
| 26 | M | 73 | MDS | M1 | + | - | - | + | + | multiple | wt | wt | TP53, CUX1 | 11.1 | 33 |
| 27 | M | 58 | Relapse | M1 | + | - | - | + | + | -7 | wt | wt | nt | 0.9 | 65 |
| 28 | F | 65 | Relapse | M6 | + | - | - | + | + | multiple | wt | wt | TP53, IKZF1, RUNX1 | 1.3 | 65 |
| 29 | M | 77 | Polycytemia vera | | - | - | - | - | + | nt | nt | nt | nt | 8.3 | 41 |
| 30 | F | 86 | *de novo* | | | | | | | del5q | wt | wt | GATA2 | 249 | 17 |
| 31 | F | 65 | Relapse | M6 | + | - | - | + | + | multiple | wt | wt | nt | 1.3 | 65 |
| 32 | F | 77 | *de novo* | M1 | - | - | - | - | - | normal | ITD | Ins | DNMT3A | 68.5 | 32 |
| 33 | M | 62 | MDS | M0 | - | - | - | - | + | multiple | nt | nt | nt | 8.2 | 57 |
| 34 | M | 68 | MDS | M0 | - | - | - | - | + | normal | wt | wt | TET2, ASXL1, BEBPA, SRSF2, STAG2 | 1.5 | 24 |
| 35 | F | 83 | *de novo* | | + | - | - | + | - | normal | ITD | Ins | nt | 105 | 38 |
| 36* | M | 78 | MDS | nt | + | - | - | + | + | nt | ITD | wt | ASXL1, STAG2, ZRSR2 | 5.6 | 55 |
| 37* | M | 62 | 3rd relapse | M1 | + | nt | - | + | + | t(4;20) | wt | wt | DNMT3, IDH2 | 1.5 | 78 |
| 38* | M | 68 | Myelofibrosis | M1 | + | - | - | + | + | Normal | wt | wt | KRAS | 34.3 | 56 |
| 39* | F | 80 | *de novo* | M2 | - | - | - | + | - | normal | ITD | Ins | PHF6, TET2, BCDFIL1, CSF3R | 217 | 5 |
| 40* | F | 70 | Chemotherapy | M4 | + | - | + | + | - | normal | wt | Ins | NRAS, DNMT3A, IDH1 | 73.7 | 7 |
| 41* | M | 60 | 2nd relapse | M4 | + | - | + | + | + | normal | ITD | wt | WT1 | 66 | 6 |
| 42 | M | 71 | Chemotherapy | M4 | | | | | | nt | wt | Ins | KRAS, DNMT3A, TET2 | 104 | 2 |
| 43 | M | 48 | Relapse | M4 | | | | | - | normal | ITD, TKD | Ins | DNMT3A, IDH1 | 30.4 | 8 |
| 44 | F | 60 | Relapse | M4 | + | - | + | + | - | normal | ITD | Ins | DNMT3A, TET2 | 16.7 | 12 |

Abbreviations: MDS, myelodysplastic syndromes; Ins, insertion; ITD, internal tandem duplications; nt, not tested; wt, wild type.

1) Patient IDs marked with (*) indicate that these patients were included in the study by Ryningen *et al.* (PMID 19007987) (n=19) [1], while unmarked patient IDs were included in the study by Fredly *et al.* (PMID 23915396) [2].

2) Expression of a marker was defined as at least 20% positive cells compared with the corresponding negative control.

3) Survival is presented as the survival from start of treatment.

**Table S2. Significantly altered serum metabolites between subsets of non-responders to antileukemic treatment based on ATRA and valproic acid; a comparison of non-responders with very aggressive (i.e. rapidly progressive) and less aggressive disease.** The arrows (↑↓) indicate whether the metabolite levels were increased or decreased in patients with very aggressive disease compared to patients with less aggressive disease.

| Main class / Subclassification | | Metabolite | *p*-value | *q*-value | Fold change, very aggressive versus less aggressive |
|---|---|---|---|---|---|
| **Amino Acid** | | | | | |
| Glycine, Serine and Threonine Metabolism | ↓ | Betaine | 0.0141 | 0.2745 | 0.75 |
| Glutamate Metabolism | ↑ | N-acetylglutamate | 0.0113 | 0.2642 | 1.51 |
| Lysine Metabolism | ↓ | 2-aminoadipate | 0.0099 | 0.2639 | 0.56 |
| Tyrosine Metabolism | ↑ | Thyroxine | 0.0185 | 0.3107 | 1.33 |
| Tryptophan Metabolism | ↑ | Tryptophan betaine | 0.0051 | 0.2185 | 6.96 |
| Methionine, Cysteine, SAM and Taurine Metabolism | ↑ | Cysteine s-sulfate | 0.0093 | 0.2587 | 1.85 |
| | ↓ | Hypotaurine | 0.0284 | 0.3801 | 0.32 |
| Urea cycle; Arginine and Proline Metabolism | ↓ | Citrulline | 0.0427 | 0.3952 | 0.70 |
| | ↑ | Dimethylarginine (SDMA + ADMA) | 0.0066 | 0.2253 | 1.69 |
| Polyamine Metabolism | ↑ | Spermidine | 0.0433 | 0.3952 | 9.72 |
| **Peptide** | | | | | |
| Dipeptide | ↓ | Isoleucylglycine | 0.0015 | 0.1292 | 0.52 |
| | ↓ | Leucylalanine | 0.0000 | 0.0022 | 0.10 |
| | ↓ | Threonylphenylalanine | 0.0371 | 0.3952 | 0.45 |
| Fibrinogen Cleavage Peptide | ↓ | Fibrinopeptide A, des-ala(1) | 0.0235 | 0.3591 | 0.27 |
| Acetylated Peptides | ↓ | Phenylacetylglycine | 0.0070 | 0.2253 | 0.15 |
| **Carbohydrate** | | | | | |
| Pentose Metabolism | ↑ | Ribonate | 0.0043 | 0.2022 | 1.40 |
| **Energy** | | | | | |
| TCA Cycle | ↓ | Aconitate [cis or trans] | 0.0351 | 0.3952 | 0.35 |
| | ↑ | Alpha-ketoglutarate | 0.0369 | 0.3952 | 1.56 |
| | ↑ | Succinate | 0.0322 | 0.3904 | 1.45 |
| | ↑ | 2-methylcitrate/homocitrate | 0.0128 | 0.2642 | 1.29 |
| **Lipid** | | | | | |
| Medium Chain Fatty Acid | ↓ | Caprate (10:0) | 0.0172 | 0.3107 | 0.57 |
| Polyunsaturated Fatty Acid (n3 and n6) | ↑ | Docosatrienoate (22:3n3) | 0.0274 | 0.3798 | 1.79 |
| | ↑ | Docosatrienoate (22:3n6) | 0.0278 | 0.3798 | 2.61 |
| Fatty Acid, Dicarboxylate | ↑ | 3-methylglutarate/2-methylglutarate | 0.0402 | 0.3952 | 1.88 |

| | | | | | |
|---|---|---|---|---|---|
| Fatty Acid Metabolism(Acyl Carnitine) | ↓ | Docosahexaenoylcarnitine (C22:6) | 0.0065 | 0.2253 | 0.31 |
| Fatty Acid Metabolism (Acyl Choline) | ↑ | Arachidonoylcholine | 0.0193 | 0.3107 | 1.38 |
| Phosphatidylcholine (PC) | ↑ | 1,2-dipalmitoyl-GPC (16:0/16:0) | 0.0377 | 0.3952 | 1.36 |
| | ↑ | 1-palmitoyl-2-palmitoleoyl-GPC (16:0/16:1) | 0.0404 | 0.3952 | 1.60 |
| | ↑ | 1-palmitoyl-2-oleoyl-GPC (16:0/18:1) | 0.0077 | 0.2253 | 1.43 |
| | ↑ | 1-palmitoyl-2-arachidonoyl-GPC (16:0/20:4n6) | 0.0326 | 0.3904 | 1.18 |
| Phosphatidylethanolamine (PE) | ↑ | 1-palmitoyl-2-linoleoyl-GPE (16:0/18:2) | 0.0074 | 0.2253 | 2.09 |
| | ↑ | 1-palmitoyl-2-arachidonoyl-GPE (16:0/20:4) | 0.0205 | 0.3211 | 2.22 |
| | ↑ | 1-palmitoyl-2-docosahexaenoyl-GPE (16:0/22:6) | 0.0033 | 0.1752 | 1.80 |
| | ↑ | 1-stearoyl-2-oleoyl-GPE (18:0/18:1) | 0.0010 | 0.1088 | 2.61 |
| | ↑ | 1-stearoyl-2-linoleoyl-GPE (18:0/18:2) | 0.0076 | 0.2253 | 1.95 |
| | ↑ | 1-stearoyl-2-arachidonoyl-GPE (18:0/20:4) | 0.0108 | 0.2642 | 1.86 |
| | ↑ | 1-stearoyl-2-docosahexaenoyl-GPE (18:0/22:6) | 0.0004 | 0.0568 | 1.60 |
| Phosphatidylinositol (PI) | ↑ | 1-stearoyl-2-linoleoyl-GPI (18:0/18:2) | 0.0191 | 0.3107 | 1.81 |
| | ↑ | 1-stearoyl-2-arachidonoyl-GPI (18:0/20:4) | 0.0434 | 0.3952 | 1.50 |
| Lysophospholipid | ↑ | 1-oleoyl-GPC (18:1) | 0.0328 | 0.3904 | 1.34 |
| | ↑ | 1-palmitoyl-GPE (16:0) | 0.0125 | 0.2642 | 1.39 |
| | ↑ | 1-stearoyl-GPE (18:0) | 0.0115 | 0.2642 | 1.49 |
| | ↑ | 2-stearoyl-GPE (18:0) | 0.0385 | 0.3952 | 1.47 |
| | ↑ | 1-stearoyl-GPG (18:0) | 0.0337 | 0.3941 | 1.71 |
| | ↑ | 1-arachidonoyl-GPI (20:4) | 0.0463 | 0.3952 | 1.47 |
| Plasmalogen | ↑ | 1-(1-enyl-palmitoyl)-2-arachidonoyl-GPC (P16:0/20:4) | 0.0189 | 0.3107 | 1.52 |
| Glycerolipid Metabolism | ↑ | Glycerophosphoglycerol | 0.0003 | 0.0568 | 1.46 |
| Monoacylglycerol | ↑ | 2-palmitoylglycerol (16:0) | 0.0422 | 0.3952 | 2.18 |
| Diacylglycerol | ↑ | Palmitoyl-linoleoyl-glycerol (16:0/18:2) [2] | 0.0426 | 0.3952 | 1.63 |
| | ↑ | Palmitoleoyl-linoleoyl-glycerol (16:1/18:2) [1] | 0.0498 | 0.3952 | 1.61 |
| | ↑ | Palmitoyl-arachidonoyl-glycerol (16:0/20:4) [2] | 0.0439 | 0.3952 | 2.12 |
| | ↑ | Oleoyl-oleoyl-glycerol (18:1/18:1) [1] | 0.0387 | 0.3952 | 2.06 |
| | ↑ | Oleoyl-oleoyl-glycerol (18:1/18:1) [2] | 0.0426 | 0.3952 | 2.25 |
| | ↑ | Oleoyl-linoleoyl-glycerol (18:1/18:2) [1] | 0.0067 | 0.2253 | 1.83 |
| | ↑ | Oleoyl-linoleoyl-glycerol (18:1/18:2) [2] | 0.0193 | 0.3107 | 1.84 |
| | ↑ | Linoleoyl-linoleoyl-glycerol (18:2/18:2) [1] | 0.0016 | 0.1292 | 1.78 |
| | ↑ | Oleoyl-arachidonoyl-glycerol (18:1/20:4) [2] | 0.0497 | 0.3952 | 2.01 |
| Sphingolipid Metabolism | ↑ | Sphinganine | 0.0008 | 0.0987 | 2.37 |

| | | | | | |
|---|---|---|---|---|---|
| | ↑ | Sphingosine | 0.0126 | 0.2642 | 2.08 |
| | ↓ | Sphingomyelin (d18:1/22:2, d18:2/22:1, d16:1/24:2) | 0.0489 | 0.3952 | 0.71 |
| Ceramides | ↑ | N-palmitoyl-sphingosine (d18:1/16:0) | 0.0028 | 0.1752 | 1.75 |
| Sterol | ↑ | Cholesterol | 0.0044 | 0.2022 | 1.46 |
| Androgenic Steroids | ↓ | 5alpha-androstan-3alpha,17beta-diol Monosulfate | 0.0494 | 0.3952 | 0.71 |
| **Nucleotide** | | | | | |
| Purine Metabolism, (Hypo)Xanthine/Inosine containing | ↑ | Xanthine | 0.0264 | 0.3798 | 3.32 |
| | ↑ | Xanthosine | 0.0344 | 0.3949 | 3.58 |
| Purine Metabolism, Adenine containing | ↓ | Adenosine 3',5'-cyclic monophosphate (cAMP) | 0.0001 | 0.0267 | 0.45 |
| | ↑ | N6-succinyladenosine | 0.0444 | 0.3952 | 1.86 |
| Purine Metabolism, Guanine containing | ↑ | 7-methylguanine | 0.0131 | 0.2642 | 1.74 |
| Pyrimidine Metabolism, Orotate containing | ↓ | Dihydroorotate | 0.0115 | 0.2642 | 0.19 |
| | ↑ | Orotidine | 0.0253 | 0.3777 | 2.20 |
| Pyrimidine Metabolism, Uracil containing | ↑ | N-acetyl-beta-alanine | 0.0481 | 0.3952 | 1.47 |
| Pyrimidine Metabolism, Cytidine containing | ↑ | 2'-O-methylcytidine | 0.0033 | 0.1752 | 1.54 |
| **Cofactors and Vitamins** | | | | | |
| Nicotinate and Nicotinamide Metabolism | ↑ | Nicotinamide | 0.0020 | 0.1425 | 7.85 |
| Tocopherol Metabolism | ↓ | Alpha-CEHC | 0.0473 | 0.3952 | 0.97 |
| Hemoglobin and Porphyrin Metabolism | ↓ | Bilirubin (E,E) | 0.0275 | 0.3798 | 0.48 |
| **Xenobiotics** | | | | | |
| Tobacco Metabolite | ↓ | Hydroxycotinine | 0.0305 | 0.3904 | 0.21 |
| Food Component/Plant | ↑ | Gluconate | 0.0296 | 0.3883 | 1.27 |
| | ↓ | Dihydroferulic acid | 0.0163 | 0.3075 | 0.18 |
| | ↓ | Umbelliferone sulfate | 0.0490 | 0.3952 | 0.40 |
| Chemical | ↓ | Methylnaphthyl sulfate | 0.0321 | 0.3904 | 0.28 |

**Table S3. Significantly altered metabolites after seven days of valproic acid monotherapy; a comparison of pretreatment samples versus samples collected during treatment for patients classified as responders to antileukemic therapy.** The comparison is based on the results for 5 responders from the study by Fredly *et al.* (PMID 23915396) [2]. Seventy-eight metabolites were significantly altered after valproic acid therapy; 48 were significantly increased and 30 decreased. The arrows (↑↓) indicate whether the metabolite was increased or decreased by the treatment. Metabolites with $q<0.05$ are marked with yellow.

| Main class / Subclassifications | | Metabolite |
|---|---|---|
| **Amino Acid (n=17)** | | |
| Glutamate Metabolism | ↑ | Glutamate |
| Lysine Metabolism | ↓ | N6,N6,N6-trimethyllysine |
| | ↑ | 2-aminoadipate |
| Phenylalanine Metabolism | ↑ | Phenylacetate |
| Tyrosine Metabolism | ↑ | 4-hydroxycinnamate sulfate |
| | ↑ | Catechol glucuronide |
| Tryptophan Metabolism | ↓ | Tryptophan |
| | ↓ | Kynurenine |
| | ↓ | Indolelactate |
| | ↓ | Indoleacetylglutamine |
| | ↓ | 5-bromotryptophan |
| Leucine, Isoleucine and Valine Metabolism | ↑ | Isovalerylcarnitine (C5) |
| | ↑ | Beta-hydroxyisovalerate |
| | ↓ | 3-methylglutarylcarnitine (2) |
| | ↑ | Isobutyrylcarnitine (C4) |
| Methionine, Cysteine, SAM and Taurine Metabolism | ↑ | Cysteine sulfinic acid |
| Urea cycle; Arginine and Proline Metabolism | ↑ | Homocitrulline |
| **Peptide (n=3)** | | |
| Dipeptide | ↑ | Glycylvaline |
| Acetylated Peptides | ↑ | Phenylacetylcarnitine |
| | ↓ | 4-hydroxyphenylacetylglutamine |
| **Carbohydrate (n=3)** | | |
| Disaccharides and Oligosaccharides | ↑ | Lactose |
| Fructose, Mannose and Galactose Metabolism | ↓ | Mannose |
| Aminosugar Metabolism | ↓ | N-acetylneuraminate |
| **Lipid (n=32)** | | |
| Medium Chain Fatty Acid | ↓ | 10-undecenoate (11:1n1) |
| | ↓ | 5-dodecenoate (12:1n7) |
| Polyunsaturated Fatty Acid (n3 and n6) | ↓ | Adrenate (22:4n6) |
| Fatty Acid, Dicarboxylate | ↑ | Adipate (C6-DC) |
| | ↑ | Suberate (C8-DC) |
| | ↑ | Sebacate (C10-DC) |
| | ↓ | 3-carboxy-4-methyl-5-pentyl-2-furanpropionate (3-Cmpfp) |
| Fatty Acid, Amino | ↓ | 2-aminooctanoate |
| Fatty Acid Metabolism (also BCAA Metabolism) | ↑ | Propionylcarnitine (C3) |
| Fatty Acid Metabolism(Acyl Glycine) | ↑ | Hexanoylglycine |
| Fatty Acid Metabolism(Acyl Carnitine) | ↑ | 3-hydroxybutyrylcarnitine (2) |
| | ↑ | Suberoylcarnitine (C8-DC) |
| | ↑ | Adipoylcarnitine (C6-DC) |
| Fatty Acid, Monohydroxy | ↓ | 2-hydroxynervonate |
| | ↑ | 3-hydroxyhexanoate |

| | | |
|---|---|---|
| | ↓ | 3-hydroxydecanoate |
| | ↓ | 3-hydroxyoleate |
| | ↓ | 3-hydroxylaurate |
| | ↑ | 5-hydroxyhexanoate |
| | ↑ | 5-hydroxyvalproate |
| Phosphatidylethanolamine (PE) | ↑ | 1-palmitoyl-2-linoleoyl-GPE (16:0/18:2) |
| | ↑ | 1-palmitoyl-2-arachidonoyl-GPE (16:0/20:4) |
| Phosphatidylinositol (PI) | ↑ | 1-palmitoyl-2-linoleoyl-GPI (16:0/18:2) |
| Diacylglycerol | ↑ | Diacylglycerol (14:0/18:1, 16:0/16:1) [1] |
| | ↑ | Diacylglycerol (14:0/18:1, 16:0/16:1) [2] |
| | ↑ | Palmitoyl-palmitoyl-glycerol (16:0/16:0) [2] |
| | ↑ | Oleoyl-oleoyl-glycerol (18:1/18:1) [2] |
| | ↓ | Linoleoyl-linolenoyl-glycerol (18:2/18:3) [1] |
| Sterol | ↑ | Beta-sitosterol |
| Androgenic Steroids | ↓ | Dehydroisoandrosterone sulfate (DHEA-S) |
| | ↓ | Androsterone glucuronide |
| Primary Bile Acid Metabolism | ↑ | Tauro-beta-muricholate |
| **Nucleotide (n=2)** | | |
| Pyrimidine Metabolism, Uracil containing | ↓ | 2'-deoxyuridine |
| Pyrimidine Metabolism, Thymine containing | ↑ | 3-aminoisobutyrate |
| **Cofactors and Vitamins (n=3)** | | |
| Ascorbate and Aldarate Metabolism | ↓ | Oxalate (ethanedioate) |
| Tocopherol Metabolism | ↑ | Gamma-CEHC |
| Hemoglobin and Porphyrin Metabolism | ↓ | Bilirubin (E,E) |
| **Xenobiotics (n=16)** | | |
| Benzoate Metabolism | ↑ | 3-(3-hydroxyphenyl)propionate sulfate |
| | ↑ | 3-(3-hydroxyphenyl)propionate |
| Food Component/Plant | ↑ | Cinnamoylglycine |
| | ↑ | Dihydroferulic acid |
| | ↑ | Ferulic acid 4-sulfate |
| | ↑ | Glycyrrhetinate |
| | ↓ | Naringenin 7-glucuronide |
| | ↓ | Isoeugenol sulfate |
| Drug - Cardiovascular | ↓ | 4-hydroxycoumarin |
| | ↓ | Candesartan |
| Drug - Neurological | ↑ | 3-hydroxyvalproate |
| | ↑ | 2-propyl-2-pentenoate (2-ene-valproate) |
| Chemical | ↑ | 3-acetylphenol sulfate |
| | ↑ | HEPES |
| | ↑ | 1,2,3-benzenetriol sulfate (2) |
| | ↑ | 2-methoxyresorcinol sulfate |
| **Partially Characterized Molecules (n=2)** | | |
| Partially Characterized Molecules | ↑ | Glucuronide of C8H16O2 (1) |
| | ↓ | Glucuronide of C14H22O4 (2) |

**Table S4. Significantly altered metabolites after seven days of valproic acid therapy; a comparison of pretreatment samples versus samples collected during treatment for patients classified as non-responders to antileukemic treatment.** The comparison is based on the results for 5 non-responders from the study by Fredly *et al.* (23915396). A total of 105 metabolites were significantly altered after valproic acid therapy; 52 were significantly increased and 53 were decreased. The arrows (↑↓) indicate whether the metabolite was increased or decreased by the treatment. Metabolites with $q$<0.05 are marked with yellow.

| Main class / Subclassifications | | Metabolite |
|---|---|---|
| **Amino Acid (n=21)** | | |
| Glycine, Serine and Threonine Metabolism | ↑ | Dimethylglycine |
| Alanine and Aspartate Metabolism | ↑ | N-acetylalanine |
| Glutamate Metabolism | ↑ | Carboxyethyl-GABA |
| | ↓ | S-1-pyrroline-5-carboxylate |
| Histidine Metabolism | ↑ | N-acetylhistidine |
| Lysine Metabolism | ↑ | 6-oxopiperidine-2-carboxylate |
| Phenylalanine Metabolism | ↑ | N-acetylphenylalanine |
| Tyrosine Metabolism | ↑ | N-acetyltyrosine |
| Tryptophan Metabolism | ↓ | Tryptophan |
| | ↑ | N-acetyltryptophan |
| | ↑ | Tryptophan betaine |
| | ↓ | Indoleacetate |
| | ↓ | Indoleacetylglutamine |
| | ↓ | 5-bromotryptophan |
| Leucine, Isoleucine and Valine Metabolism | ↑ | Beta-hydroxyisovalerate |
| | ↓ | 3-methylglutarylcarnitine (2) |
| | ↑ | Ethylmalonate |
| | ↑ | Isobutyrylcarnitine (C4) |
| | ↑ | 2,3-dihydroxy-2-methylbutyrate |
| Methionine, Cysteine, SAM and Taurine Metabolism | ↑ | N-acetylmethionine |
| Urea cycle; Arginine and Proline Metabolism | ↑ | Trans-4-hydroxyproline |
| **Peptide (n=2)** | | |
| Acetylated Peptides | ↓ | 4-hydroxyphenylacetylglutamine |
| | ↓ | Phenylacetylglycine |
| **Carbohydrate (n=4)** | | |
| Pentose Metabolism | ↑ | Ribitol |
| | ↑ | Xylose |
| | ↑ | Arabitol/xylitol |
| Fructose, Mannose and Galactose Metabolism | ↓ | Galactonate |
| **Energy (n=1)** | | |
| TCA Cycle | ↓ | Succinylcarnitine (C4-DC) |
| **Lipid (n=49)** | | |
| Medium Chain Fatty Acid | ↓ | Caprate (10:0) |
| | ↓ | 10-undecenoate (11:1n1) |
| Fatty Acid, Dicarboxylate | ↑ | Adipate (C6-DC) |
| | ↑ | Pimelate (C7-DC) |
| | ↑ | Suberate (C8-DC) |
| | ↓ | Dodecanedioate (C12-DC) |

| | | |
|---|---|---|
| | ↓ | Tetradecanedioate (C14-DC) |
| | ↓ | Hexadecenedioate (C16:1-DC) |
| | ↓ | Octadecanedioate (C18-DC) |
| | ↓ | Octadecenedioate (C18:1-DC) |
| | ↓ | Eicosanodioate (C20-DC) |
| | ↓ | 3-carboxy-4-methyl-5-pentyl-2-furanpropionate (3-Cmpfp) |
| Fatty Acid Metabolism(Acyl Glycine) | ↑ | Hexanoylglycine |
| Fatty Acid Metabolism(Acyl Carnitine) | ↑ | Hexanoylcarnitine (C6) |
| | ↑ | 5-dodecenoylcarnitine (C12:1) |
| | ↑ | Cis-4-decenoylcarnitine (C10:1) |
| | ↑ | Laurylcarnitine (C12) |
| | ↑ | Myristoylcarnitine (C14) |
| | ↑ | Myristoleoylcarnitine (C14:1) |
| | ↑ | Suberoylcarnitine (C8-DC) |
| | ↑ | Adipoylcarnitine (C6-DC) |
| | ↑ | Pimeloylcarnitine/3-methyladipoylcarnitine (C7-DC) |
| Fatty Acid, Monohydroxy | ↓ | 2-hydroxyoctanoate |
| | ↓ | 2-hydroxydecanoate |
| | ↑ | 3-hydroxyhexanoate |
| | ↑ | 3-hydroxysebacate |
| | ↑ | 5-hydroxyvalproate |
| Endocannabinoid | ↓ | N-stearoylserine |
| Phosphatidylcholine (PC) | ↓ | 1-stearoyl-2-docosahexaenoyl-GPC (18:0/22:6) |
| Phosphatidylinositol (PI) | ↑ | 1-palmitoyl-2-linoleoyl-GPI (16:0/18:2) |
| | ↑ | 1-stearoyl-2-linoleoyl-GPI (18:0/18:2) |
| Lysophospholipid | ↓ | 1-palmitoyl-GPC (16:0) |
| | ↓ | 1-palmitoleoyl-GPC (16:1) |
| | ↓ | 1-stearoyl-GPC (18:0) |
| Diacylglycerol | ↓ | Diacylglycerol (14:0/18:1, 16:0/16:1) [1] |
| | ↓ | Diacylglycerol (14:0/18:1, 16:0/16:1) [2] |
| | ↓ | Palmitoyl-oleoyl-glycerol (16:0/18:1) [2] |
| | ↓ | Palmitoyl-arachidonoyl-glycerol (16:0/20:4) [2] |
| Sphingolipid Metabolism | ↓ | Sphinganine-1-phosphate |
| Progestin Steroids | ↓ | 5alpha-pregnan-3beta,20alpha-diol monosulfate (2) |
| | ↓ | Pregnanediol-3-glucuronide |
| Corticosteroids | ↑ | Cortisone |
| Androgenic Steroids | ↑ | Androstenediol (3beta,17beta) disulfate (1) |
| | ↑ | Androstenediol (3beta,17beta) disulfate (2) |
| | ↑ | Andro steroid monosulfate C19H28O6S (1) |
| Primary Bile Acid Metabolism | ↓ | Glycochenodeoxycholate glucuronide (1) |
| Secondary Bile Acid Metabolism | ↓ | Isoursodeoxycholate |
| | ↓ | 7-ketolithocholate |
| | ↓ | 3b-hydroxy-5-cholenoic acid |
| **Nucleotide (n=6)** | | |
| Purine Metabolism, (Hypo)Xanthine/Inosine containing | ↑ | N1-methylinosine |
| Purine Metabolism, Adenine containing | ↑ | N6-carbamoylthreonyladenosine |

| | | |
|---|---|---|
| Purine Metabolism, Guanine containing | ↑ | 7-methylguanine |
| Pyrimidine Metabolism, Uracil containing | ↑ | 3-ureidopropionate |
| Pyrimidine Metabolism, Cytidine containing | ↑ | Cytidine |
| Pyrimidine Metabolism, Thymine containing | ↑ | 3-aminoisobutyrate |
| **Cofactors and Vitamins (n=4)** | | |
| Nicotinate and Nicotinamide Metabolism | ↑ | N1-Methyl-2-pyridone-5-carboxamide |
| Pantothenate and CoA Metabolism | ↓ | Pantothenate |
| Tocopherol Metabolism | ↑ | Gamma-CEHC |
| Hemoglobin and Porphyrin Metabolism | ↓ | Bilirubin (E,E) |
| **Xenobiotics (n=15)** | | |
| Benzoate Metabolism | ↓ | 4-hydroxyhippurate |
| | ↓ | Catechol sulfate |
| | ↓ | Guaiacol sulfate |
| | ↓ | 4-ethylphenylsulfate |
| Xanthine Metabolism | ↑ | 3-methylxanthine |
| Food Component/Plant | ↓ | Ferulylglycine (1) |
| | ↓ | Ferulylglycine (2) |
| | ↓ | Acesulfame |
| | ↓ | Thymol sulfate |
| | ↓ | 4-allylphenol sulfate |
| Drug - Analgesics, Anesthetics | ↓ | Lidocaine |
| Drug - Cardiovascular | ↓ | 4-hydroxycoumarin |
| Drug - Neurological | ↑ | 3-hydroxyvalproate |
| | ↑ | 2-propyl-2-pentenoate (2-ene-valproate) |
| Chemical | ↑ | O-sulfo-L-tyrosine |
| **Partially Characterized Molecules (n=3)** | | |
| Partially Characterized Molecules | ↑ | Glucuronide of C8H16O2 (1) |
| | ↓ | Glucuronide of C10H18O2 (7) |
| | ↓ | Glucuronide of C14H22O4 (2) |

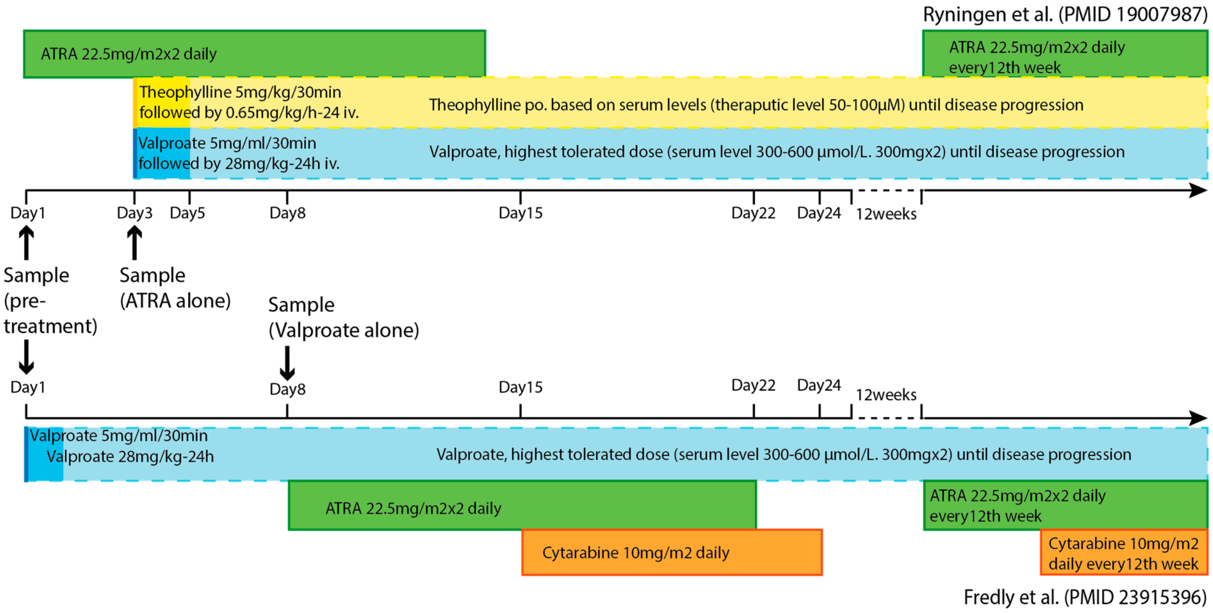


**Figure S1. Timeline of treatment schedule for patients included in two clinical studies.** In the first study shown at the top (Ryningen *et al.*), patients were give ATRA alone for two days before theophylline and valproic acid were given on day 3. Samples were collected pretherapy (day 1) and after 2-days of ATRA treatment. In the second study shown at the bottom (Fredly *et al.*), patients received valproic acid alone for 7 days before ATRA and then subsequently cytarabine were given. Samples were collected pretherapy (day 1) and after 7 days of valproic acid therapy.

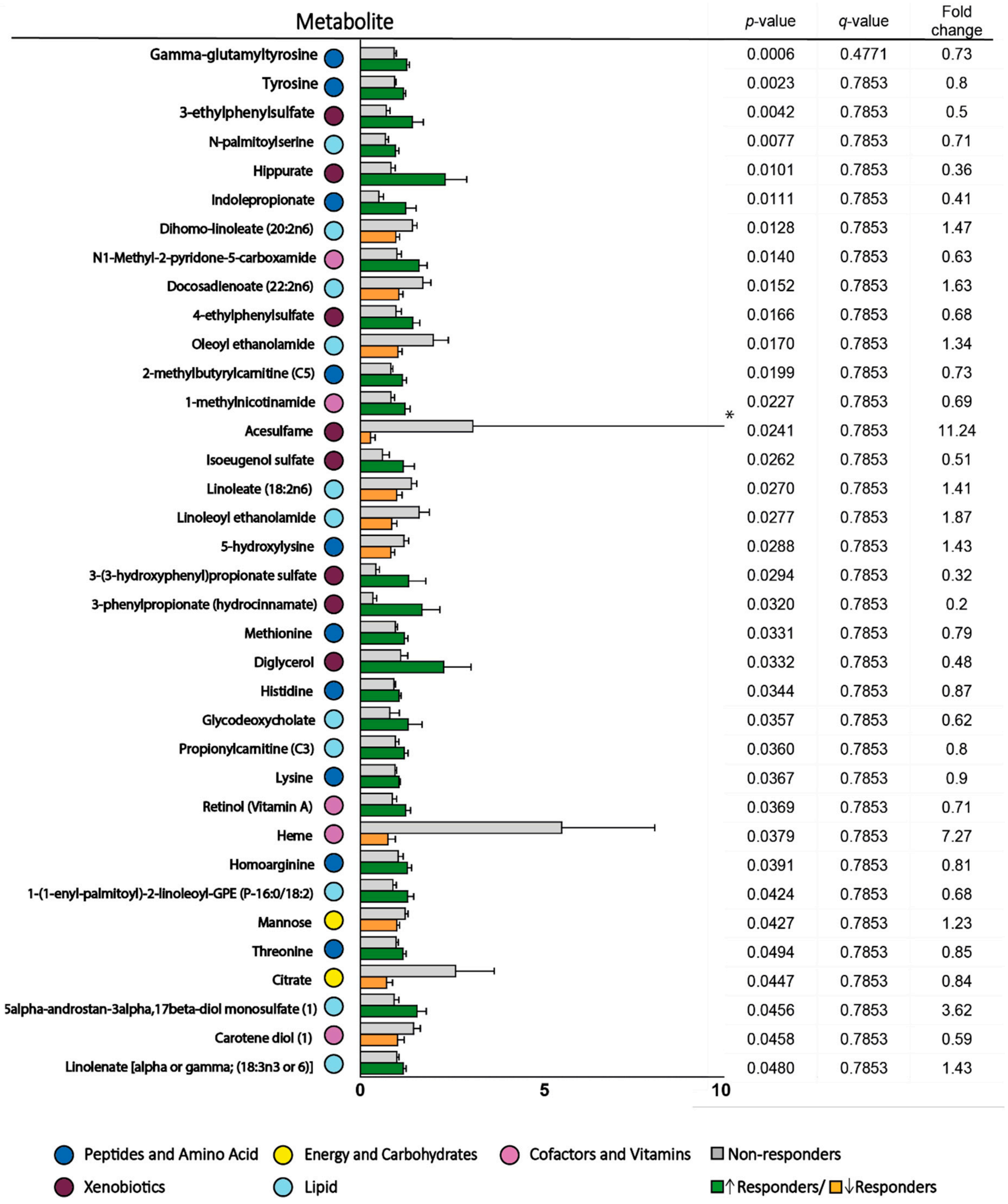


| Metabolite | *p*-value | *q*-value | Fold change |
|---|---|---|---|
| Gamma-glutamyltyrosine | 0.0006 | 0.4771 | 0.73 |
| Tyrosine | 0.0023 | 0.7853 | 0.8 |
| 3-ethylphenylsulfate | 0.0042 | 0.7853 | 0.5 |
| N-palmitoylserine | 0.0077 | 0.7853 | 0.71 |
| Hippurate | 0.0101 | 0.7853 | 0.36 |
| Indolepropionate | 0.0111 | 0.7853 | 0.41 |
| Dihomo-linoleate (20:2n6) | 0.0128 | 0.7853 | 1.47 |
| N1-Methyl-2-pyridone-5-carboxamide | 0.0140 | 0.7853 | 0.63 |
| Docosadienoate (22:2n6) | 0.0152 | 0.7853 | 1.63 |
| 4-ethylphenylsulfate | 0.0166 | 0.7853 | 0.68 |
| Oleoyl ethanolamide | 0.0170 | 0.7853 | 1.34 |
| 2-methylbutyrylcarnitine (C5) | 0.0199 | 0.7853 | 0.73 |
| 1-methylnicotinamide | 0.0227 | 0.7853 | 0.69 |
| Acesulfame | 0.0241 | 0.7853 | 11.24 |
| Isoeugenol sulfate | 0.0262 | 0.7853 | 0.51 |
| Linoleate (18:2n6) | 0.0270 | 0.7853 | 1.41 |
| Linoleoyl ethanolamide | 0.0277 | 0.7853 | 1.87 |
| 5-hydroxylysine | 0.0288 | 0.7853 | 1.43 |
| 3-(3-hydroxyphenyl)propionate sulfate | 0.0294 | 0.7853 | 0.32 |
| 3-phenylpropionate (hydrocinnamate) | 0.0320 | 0.7853 | 0.2 |
| Methionine | 0.0331 | 0.7853 | 0.79 |
| Diglycerol | 0.0332 | 0.7853 | 0.48 |
| Histidine | 0.0344 | 0.7853 | 0.87 |
| Glycodeoxycholate | 0.0357 | 0.7853 | 0.62 |
| Propionylcarnitine (C3) | 0.0360 | 0.7853 | 0.8 |
| Lysine | 0.0367 | 0.7853 | 0.9 |
| Retinol (Vitamin A) | 0.0369 | 0.7853 | 0.71 |
| Heme | 0.0379 | 0.7853 | 7.27 |
| Homoarginine | 0.0391 | 0.7853 | 0.81 |
| 1-(1-enyl-palmitoyl)-2-linoleoyl-GPE (P-16:0/18:2) | 0.0424 | 0.7853 | 0.68 |
| Mannose | 0.0427 | 0.7853 | 1.23 |
| Threonine | 0.0494 | 0.7853 | 0.85 |
| Citrate | 0.0447 | 0.7853 | 0.84 |
| 5alpha-androstan-3alpha,17beta-diol monosulfate (1) | 0.0456 | 0.7853 | 3.62 |
| Carotene diol (1) | 0.0458 | 0.7853 | 0.59 |
| Linolenate [alpha or gamma; (18:3n3 or 6)] | 0.0480 | 0.7853 | 1.43 |

**Figure S2. Identification and classification of metabolites in pretherapy serum samples that differed significantly between responders and non-responders to the antileukemic treatment of ATRA plus valproic acid.** Thirty-six metabolites differed significantly between responders and non-responders ($p<0.05$, Welch´s two sample *t*-test). The *p*-values, *q*-values and mean fold change values for each metabolite are listed to the right in the figure (ranked by *p*-value), and a fold change >1 indicates that the levels were increased in responders compared with non-responders. Metabolite levels for non-responders are shown in grey, while increased levels in responders are shown in green (25/36 increased), and decreased levels in responders are shown in orange (11/36 decreased). Color codes for classification of metabolites are explained at the bottom of the figure. Error bars show Standard deviation (SD). * Acesulfame SD 8.963.

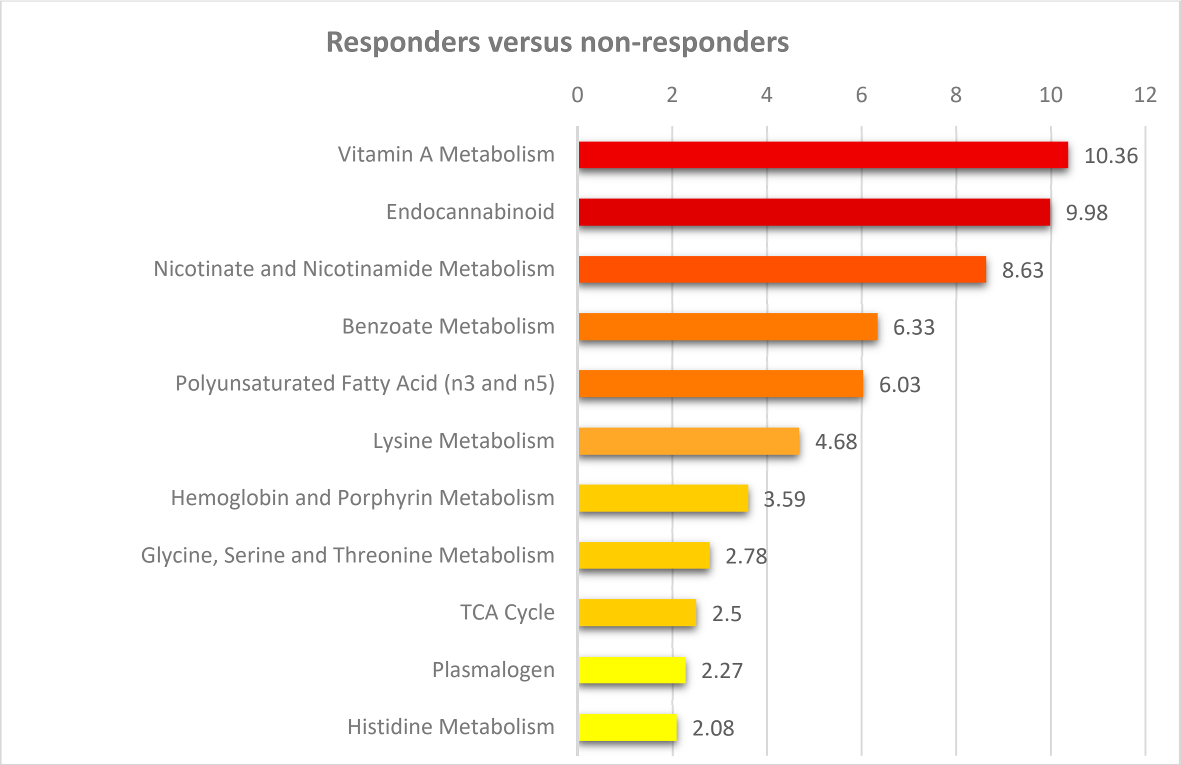


**Figure S3. Pathway enrichment analysis based on pretreatment levels of metabolites that differed between responders and non-responders to antileukemic treatment based on ATRA and valproic acid.** The analysis is used for visualization and biological interpretation of the metabolomics data and was based on significantly altered metabolites ($p<0.05$). Only signaling pathways with an enrichment value greater than two and at least two metabolites within each pathway are shown. The most significant pathway is shown in red and less significant pathways in light yellow.

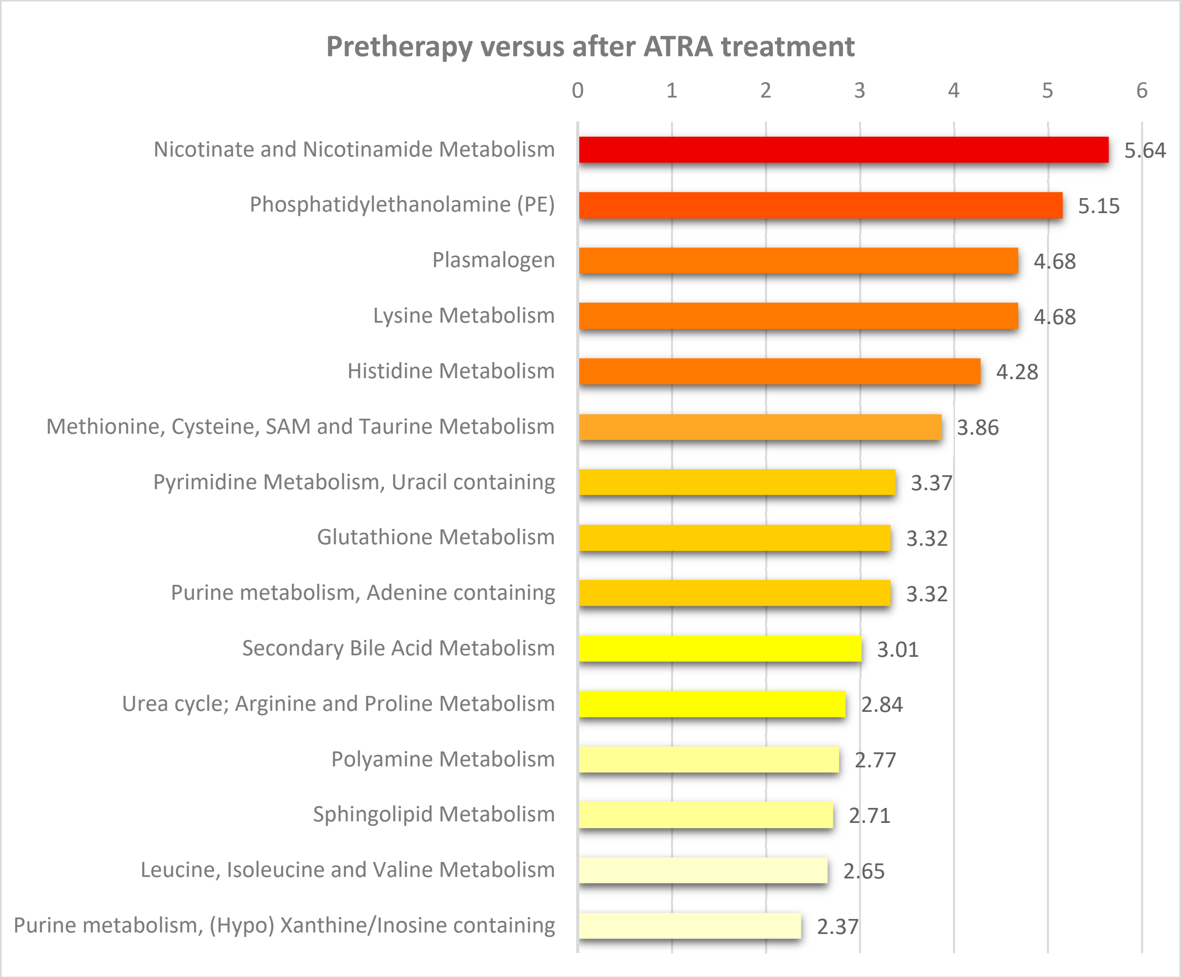


**Figure S4. Pathway enrichment analysis based on metabolite levels that differed in patients pretherapy compared to during ATRA treatment for responders and non-responders to antileukemic treatment.** The analysis was based on significantly altered metabolites ($p<0.05$), and only signaling pathways with an enrichment value greater than two and at least two metabolites within each pathway are shown in the figure. The most significant pathway is shown in red and less significant pathways in light yellow.

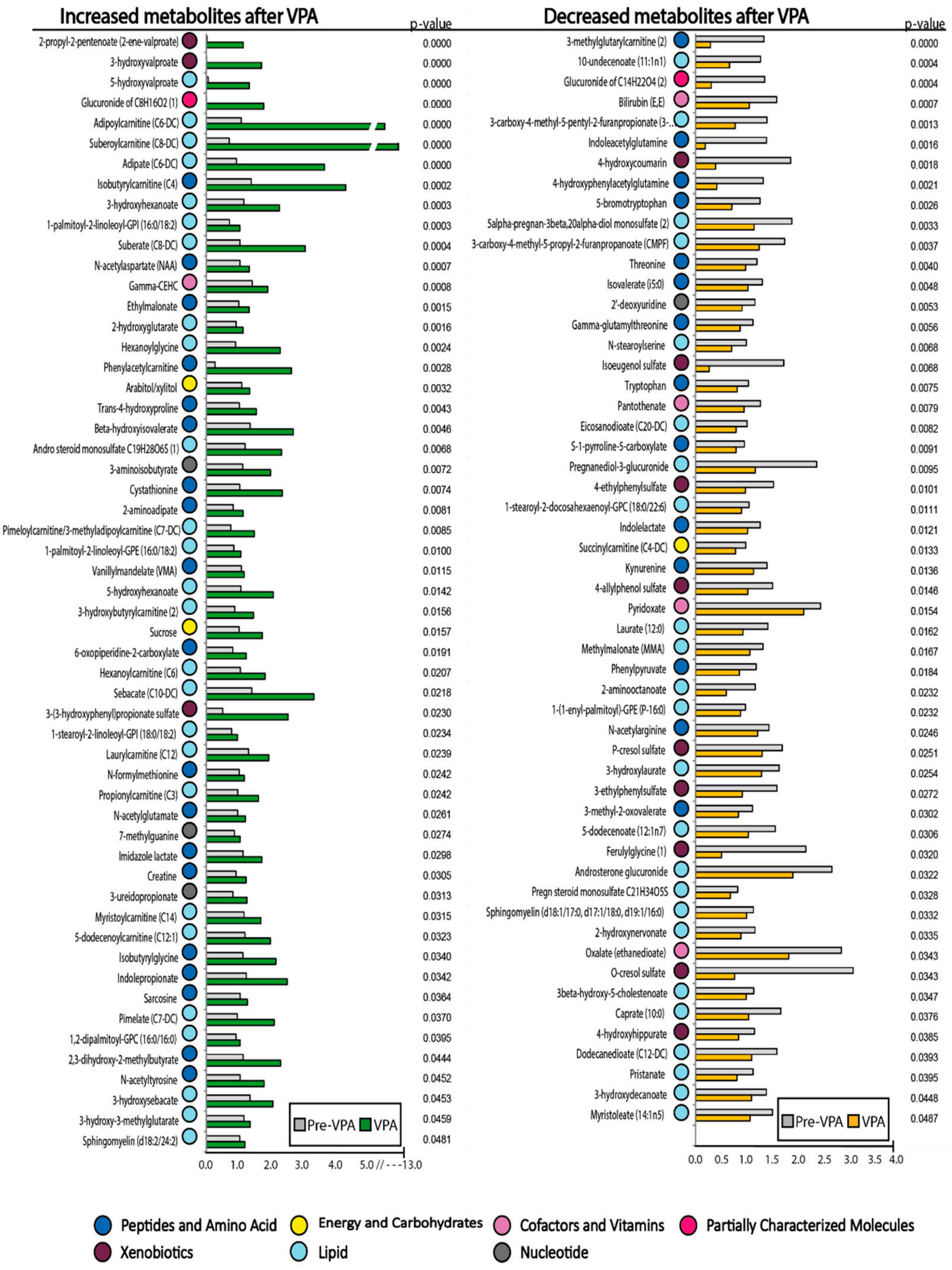


**Supplementary Figure 5. The effect of 7-day valproic acid (VPA) monotherapy on the serum metabolomic profiles of AML patients.** A total of 109 metabolites were significantly altered after VPA treatment ($p < 0.05$). The *p*-values for each individual metabolite are listed to the right, ranked according to *p*-values. Pretherapy systemic levels of metabolites are presented in light grey, increased levels during VPA treatment are presented in green and decreased levels in yellow. Color codes for classification of individual metabolites are explained at the bottom of the figure.

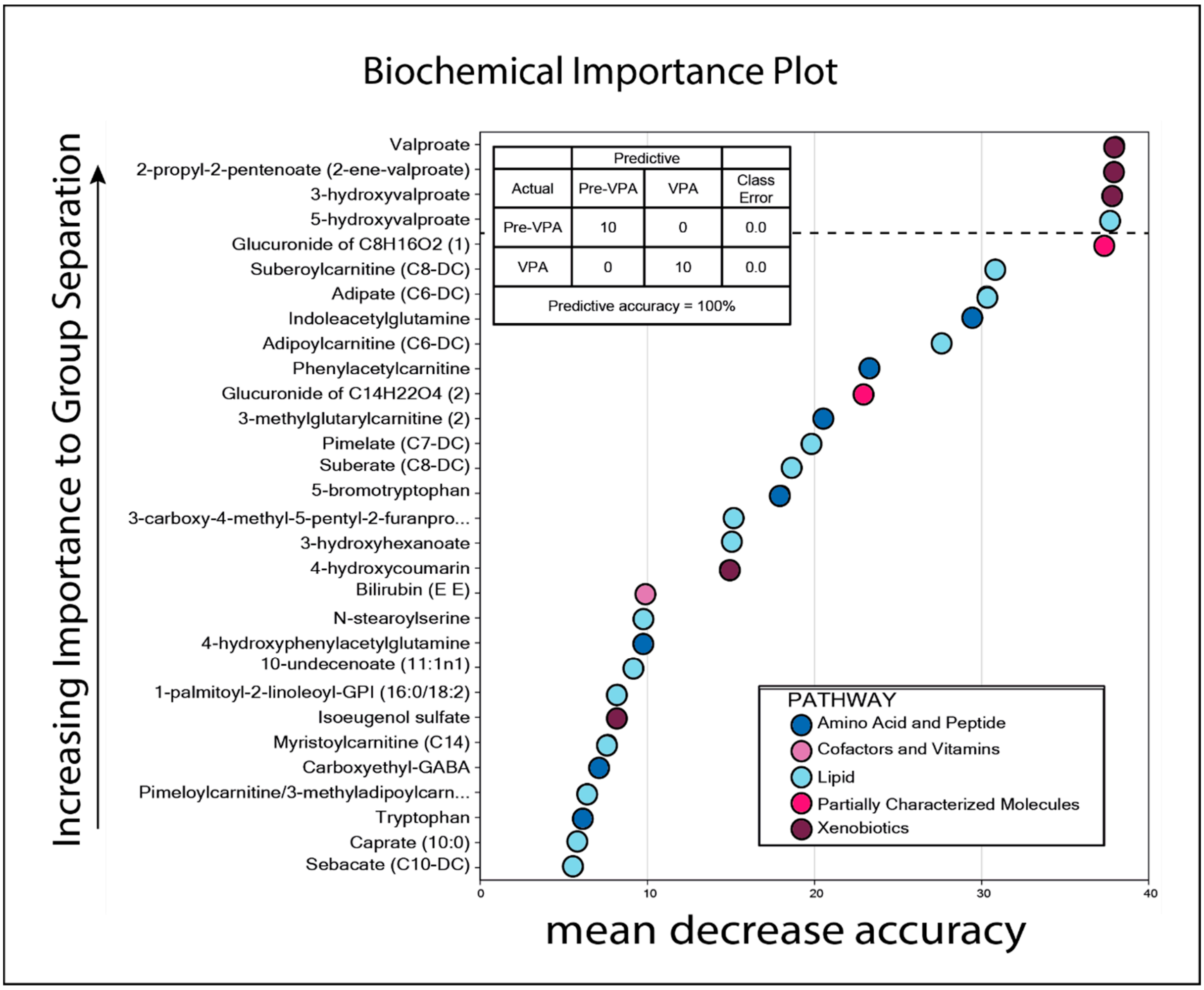


| | Predictive | | |
|---|---|---|---|
| Actual | Pre-VPA | VPA | Class Error |
| Pre-VPA | 10 | 0 | 0.0 |
| VPA | 0 | 10 | 0.0 |
| Predictive accuracy = 100% | | | |

**Supplementary Figure 6. The effect of valproic acid monotherapy for seven days on the serum metabolomics profiles for 10 patients (5 responders and 5 non-responders; patients included in the study described in PMID 23915396, valproic acid metabolites included in the study).** The random forest analysis was based on the identification of all 886 metabolites in pretherapy samples and samples collected after seven days of treatment. The analysis showed a predictive accuracy of 100% (insert table). The importance plot shows the metabolites listed after their importance for separation of the two sets of samples. The figure shows the top-30 ranked metabolites. Color codes for classification of metabolites are shown to the lower right.

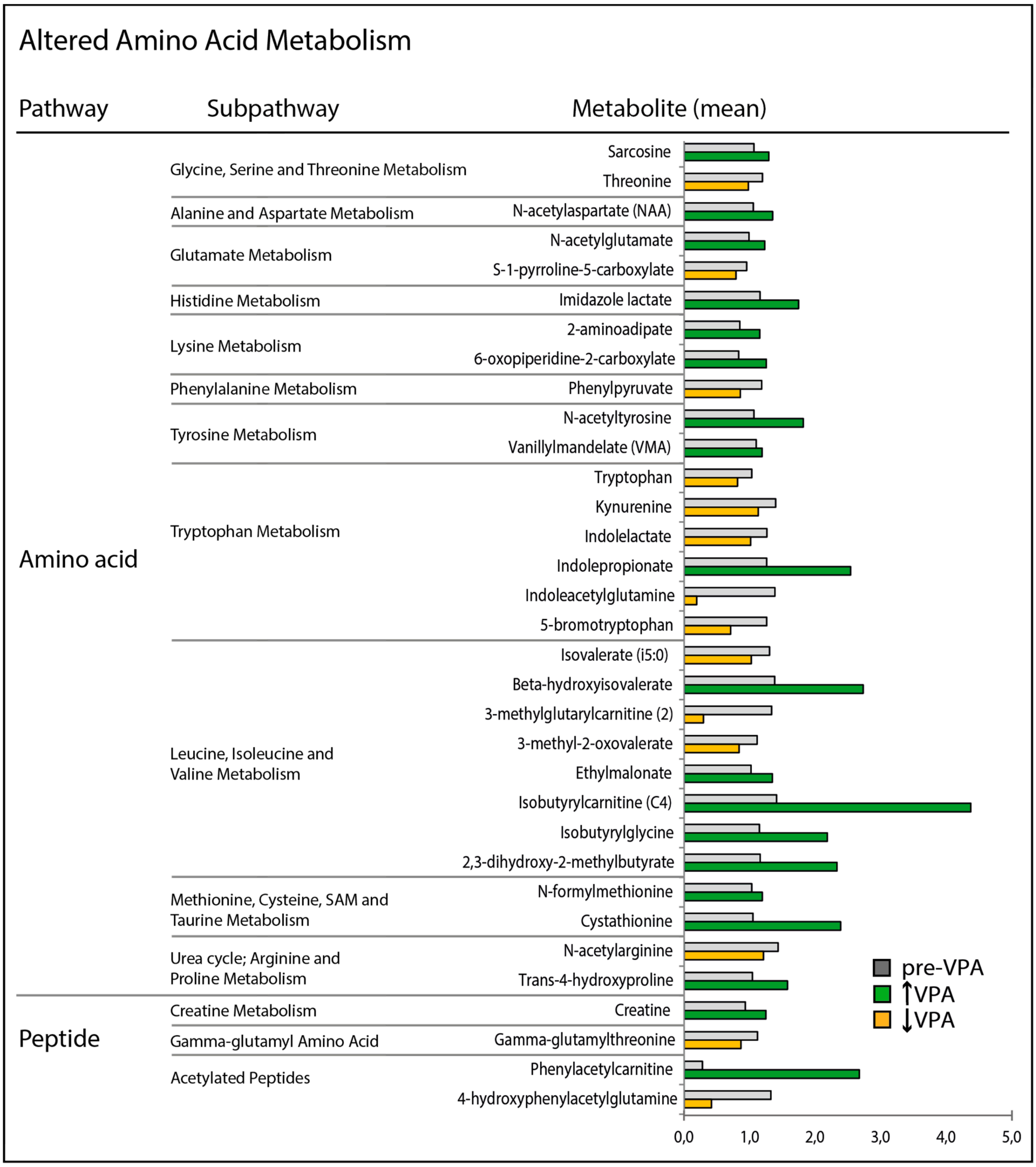


**Supplementary Figure 7. The effect of 7-day valproic acid monotherapy on serum metabolomic profiles; significantly altered amino acid and peptide metabolites when comparing samples derived from 10 patients (5 responders and 5 non-responders; all patients included in the study by Fredly et al. PMID 23915396)**. The pretreatment samples are presented in grey; metabolites increased during valproic acid therapy are presented in green and decreased levels after valproic acid therapy are presented in yellow.

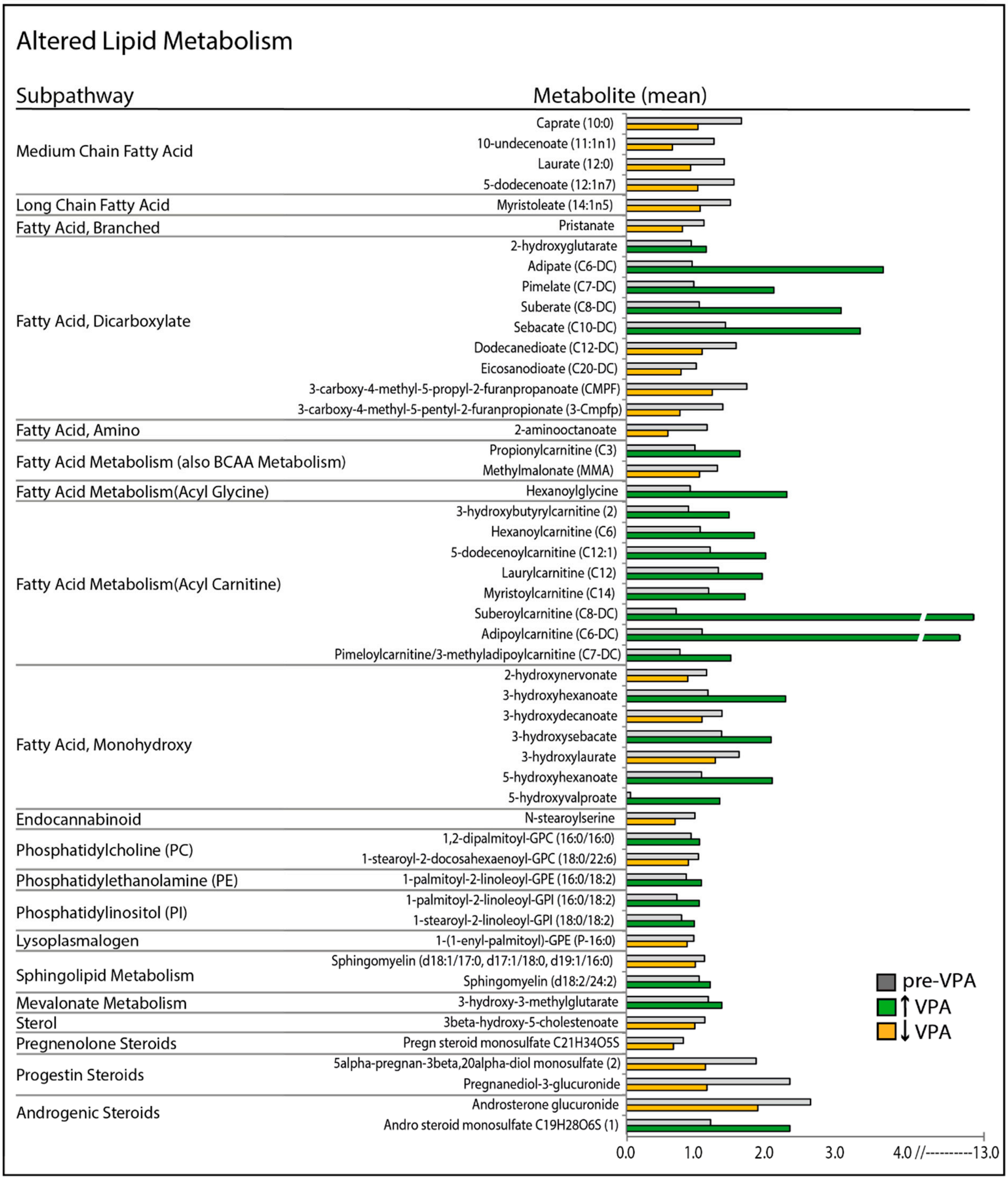


**Supplementary Figure 8. The effect of 7-day valproic acid monotherapy on serum metabolomic profiles; significantly altered lipid metabolites when comparing samples derived from 10 patients (5 responders and 5 non-responders; all patients included in the study by Fredly et al. PMID 23915396).** The pretreatment samples are presented in grey; metabolites increased during valproic acid therapy are presented in green and decreased levels after valproic acid treatment are presented in yellow.
